# Biomaterials and Cell Therapy Combination in Central Nervous System Treatments

**DOI:** 10.1021/acsabm.3c01058

**Published:** 2023-12-29

**Authors:** Zoe Giorgi, Valeria Veneruso, Emilia Petillo, Pietro Veglianese, Giuseppe Perale, Filippo Rossi

**Affiliations:** † Department of Chemistry, Materials and Chemical Engineering “Giulio Natta”, Politecnico di Milano, piazza Leonardo da Vinci 32, 20133, Milan, Italy; ‡ Istituto di Ricerche Farmacologiche Mario Negri IRCCS, Via Mario Negri 2, 20156 Milan, Italy; § Faculty of Biomedical Sciences, University of Southern Switzerland (USI), Via Buffi 13, 6900 Lugano, Switzerland; ∥ Ludwig Boltzmann Institute for Experimental and Clinical Traumatology, Donaueschingenstrasse 13, 1200 Vienna, Austria

**Keywords:** biomaterials, central nervous system, hydrogels, polymers, tissue engineering

## Abstract

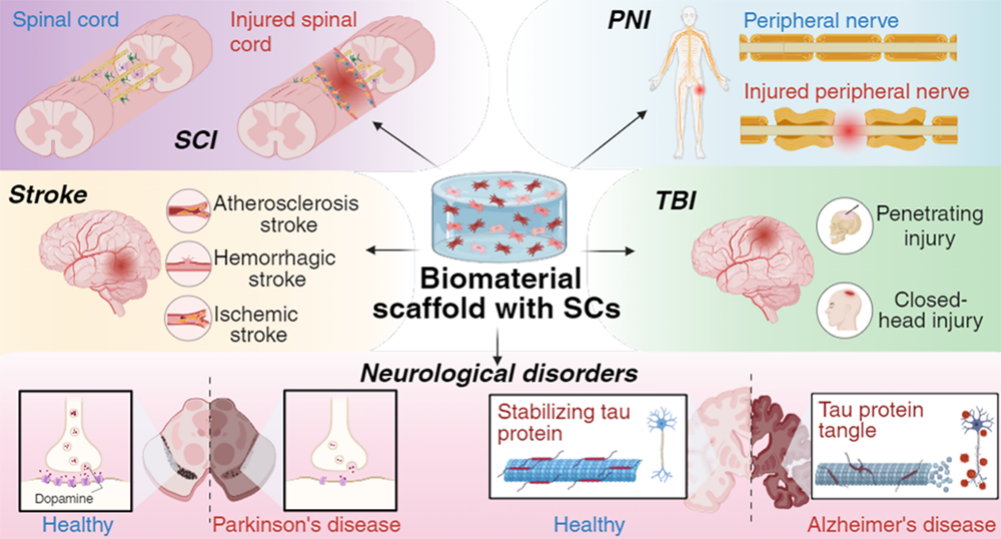

Current pharmacological and surgical therapies for the central nervous system (CNS) show a limited capacity to reduce the damage progression; that together with the intrinsic limited capability of the CNS to regenerate greatly reduces the hopes of recovery. Among all the therapies proposed, the tissue engineering strategies supplemented with therapeutic stem cells remain the most promising. Neural tissue engineering strategies are based on the development of devices presenting optimal physical, chemical, and mechanical properties which, once inserted in the injured site, can support therapeutic cells, limiting the effect of a hostile environment and supporting regenerative processes. Thus, this review focuses on the employment of hydrogel and nanofibrous scaffolds supplemented with stem cells as promising therapeutic tools for the central and peripheral nervous systems in preclinical and clinical applications.

## 1 Introduction

Physical injuries to the central nervous system (CNS) and neurodegenerative diseases damage the brain or the spinal cord, resulting in cellular degeneration and death with a consequent loss of function. The CNS has a limited regenerative capacity since the replacement of neurons is hampered by the inflammatory response and glial scar formation after the injury. Moreover, pharmacological treatments show limited effects due to the physical and chemical barriers within the CNS that favor a fast clearance, and some drugs are characterized by harmful side effects. Surgical approaches, on the other hand, can lead to further complications so that a minority of patients can really get benefits. In this context, stem cell therapy can be useful to repopulate the nervous tissue by implanting stem cells and guiding their differentiation into neurons. However, the aggressive injury environment and the tendency of cells to leave the injury site if not confined by a support reduce the performances of this approach. Thus, the combination of scaffolds and cells should be the optimal strategy for tissue regeneration since the porous structure of scaffolds provides physical support for cell adhesion, growth, and proliferation. This review initially focuses on the biomaterials options for scaffold production, dividing them into the two categories of hydrogel-based and nanofibrous, and on the most used stem cell typologies. Furthermore, a report of some preclinical applications of this combined strategy for the treatment of central and peripheral nervous system injuries and diseases is presented in order to show the benefits and possible clinical applicability.

## 2 Biomaterials for Regeneration Strategies

Tissue engineering approaches are based on the combination of three-dimensional scaffolds with living cells, and/or biologically active molecules, such as drugs or growth factors, forming a construct able to promote the repair and/or regeneration of tissues and organs.^1^

**Figure 1:**
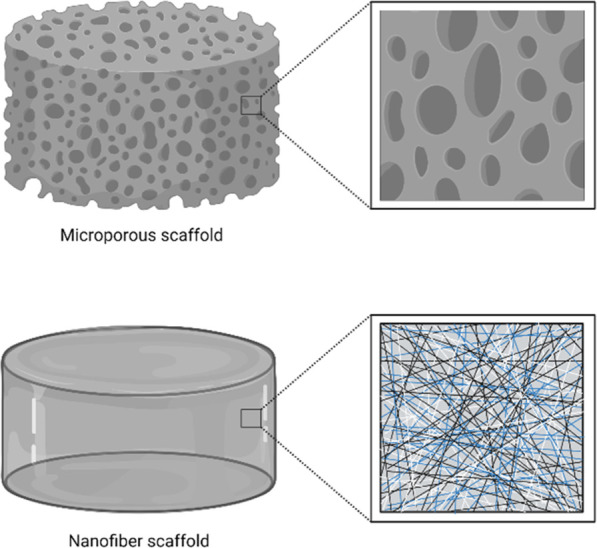
Scaffold architecture. Differences between microporous scaffold and nanofiber scaffold.

In this context, scaffolds are required to be biocompatible (not to produce an unfavorable physiological response) and biodegradable (to get eliminated from the body via naturally occurring processes), and their degradation rates should kinetically match with the evolving environment for a successful regeneration process.^2,3^ Furthermore, a scaffold’s properties must be tuned to provide physical support for cell adhesion and proliferation, while respecting the chemical and mechanical properties of native tissues. For both these reasons, scaffolds can be defined as “biomimetic materials” presenting a porous structure with an interconnected pore network useful for the formation of tissues with good spatial and temporal control.^4,5^ Tissue engineering scaffolds can be composed of different types of materials; however, polymeric biomaterials and composites are the most used ones because of their ease of handling and property modeling.^2^ Among the polymeric materials, hydrogels and nanofibers are largely employed as scaffolds (Figure 1), and their most important characteristics are summarized in Table 1.

**Table 1:** Classification of Scaffolds with Their Most Important Characteristics

Hydrogels		
natural-based	synthetic	in nervous tissue
advantages:	advantages:	• retention of peptides and extracellular proteins stimulating axonal growth and myelination
• low inflammatory responses	• controllable chemical and physical properties	• neurotrophic growth factors and drug release
• biocompatibility and biodegradability	• well-defined mechanical and degradation properties	• conductive hydrogels can enhance the regeneration process through electrical stimulation
• low cost	• large scale productions	
• easy extraction and synthesis	• low batch-to-batch variation	
• low toxicity and nontoxic degradation	• stimuli responsiveness	
• mechanical properties similar to those of living tissues		
disadvantages:	disadvantages:	
• difficult processability	• limited biocompatibility and biodegradability	
• nonoptimal mechanical properties	• possible toxicity	
• batch-to-batch variability		
• possible too high degradation rate		

Nanofibers		
electrospinning	molecular self-assembling	in nervous tissue
advantages:	advantages:	• aligned fibers guiding axonal growth
• fabrication of biomimetic structures similar to the scale and morphology of ECM	• easy processability	• neurotrophic factors and drugs release
• cost-effective approach	• massive production is possible	• electrical stimulation is possible to enhance the regeneration process
• nanofiber properties can be tailored for the specific tissue application		• SAP degradation products can enhance repair and regeneration
• high surface area		• glial scar inhibiting SAP scaffolds
disadvantages:	disadvantages:	
• low porosity	• lower ability to control the scale of the resulting fibers	
• not applicable on all types of polymers	• time-consuming process	
• poor loading efficiency and low porosity	• only lab scale production	
• possible unstable structures	• not applicable on all types of polymers	

“*Hydrogels* are three-dimensional networks of hydrophilic polymers held together by covalent, ionic or hydrogen bonds and, in the presence of solvents, they are able to swell, maintaining their original shape forming elastic gels.”^6^ Moreover, hydrogels are biocompatible and biodegradable soft biomimetic materials characterized by high water content that simulates the aqueous microenvironment of human tissues. Depending on the application, properties such as swelling behavior, polymeric mesh size, and degradation rate can be tailored by properly modifying the polymer composition or the cross-linking density.^7−9^ Furthermore, their ability to retain peptides, extracellular proteins, and growth factors stimulating axonal growth and myelination is extremely fascinating for nervous tissue applications.^10,11^ In addition, hydrogels characterized by electroconductive properties can stimulate neuron growth through electrical signal transmission.^11^

Although only a few polymers can be hydrogel backbones due to biocompatibility requirements, it is possible to distinguish natural-based and synthetic hydrogels.^12^ Collagen, gelatin, hyaluronic acid, agarose, chitosan, and alginate are common natural hydrogels which show properties extremely similar to the ones of living tissues, although they are characterized by difficult processability, nonoptimal mechanical properties, and batch-to-batch variability.^7,13^

To overcome these limitations, synthetic polymers such as acrylic polymers, poly(ethylene glycol) (PEG), poly(vinyl alcohol) (PVA), and poly(ethylene oxide) (PEO) with well-defined chemical, physical, and mechanical properties can be chosen, but they can show limited biocompatibility and biodegradability and potential toxicity.^14^ Moreover, synthetic hydrogels in which the polymeric networks are endowed with functional groups that make the gel responsive to physical, chemical, or biochemical stimuli are nowadays extremely popular materials called stimuli-responsive or smart hydrogels.^15−18^ Lastly, it is possible to combine via physical or chemical means natural and synthetic polymers, obtaining hybrid hydrogels presenting the desired bioactivity, biocompatibility, and mechanical properties.^19,20^

*Nanofiber scaffolds* are based on the idea of fabricating biomimetic structures similar to the scale and morphology of the native extracellular matrix (ECM), which is a nanofiber gel network composed of a meshwork of structural proteins, such as collagen and elastin, and nonstructural proteins, like glycosaminoglycans. The diameter of the ECM structural fibers is between 50 and 300 nm, and the fibers provide anchoring points for cell attachment while maintaining the overall tissue or organ shape and form.^21^ Thus, nanofiber scaffolds are characterized by a nanoscale diameter, high surface area/volume ratio, and high porosity with interconnected pores providing a large surface area for cell attachment and sufficient space for nutrient and waste exchange.^22^ Moreover, nanofiber scaffolds show low levels of toxicity, excellent ability to deliver their encapsulated substances to the target site avoiding side effects, stability, sterility, flexibility, and processability.^23^ Until now, a variety of approaches have been developed for fabricating this type of scaffold, such as temperature-induced phase separation, molecular self-assembly, template synthesis, drawing, and electrospinning.^24^

Among these, electrospinning is the most widely used and successful technique used since it is a cost-effective versatile approach tailorable for the specific tissue application. Most biocompatible synthetic and natural polymers can be electrospun into nanofibers independently or as blends of multiple polymers by passing through the high voltage (10–20 kV) of an electrospinning machine, leading to the formation of fine fibers with nanoscale diameters.^21,23^ Common tissue engineering nanofibrous scaffolds are composed of chitosan, silk fibroin, collagen, gelatin, poly(vinyl alcohol) (PVA), poly(l-lactide) (PLLA), polycaprolactone (PCL), poly(l-lactide-*co*-caprolactone) (PLCL), and poly(lactide-*co*-glycolide) (PLGA).^25,26^ Electrospun polymers are appealing for nervous tissue engineering approaches owing to the possibility of obtaining scaffolds with aligned fibers which can guide axonal extension toward designated targets, reforming synaptic connections and helping in the nerve function restoration process.^27^ Moreover, electrospun nanofibers can be a promising delivery vehicle for neurotrophic factors and anti-inflammation drugs.^28^ Furthermore, nanofibers fabricated using conducting polymers (i.e., polymers with loose electrons in their skeletons) can stimulate neuron growth through electrical signal transmission.^28^

Molecular self-assembling is a remarkable technique as well. Self-assembling can be defined as “the spontaneous association of molecules under equilibrium conditions into stable and structurally well-defined aggregates joined by noncovalent bonds”,^29^ and it is common for nucleotides and peptides.^2^ In particular, self-assembling peptides (SAPs) have been intensively studied after their discovery in the early 1990s^30−32^ due to their simple synthesis, functionalization, and property modification.^33^ Moreover, SAPs are characterized by unique features making them optimal scaffold choices since they are synthetic materials composed of natural building blocks forming well-organized nanofibrous structures able to retain an enormous amount of water, similarly to hydrogels.^34,35^ Furthermore, these peptide molecules can break down into nontoxic and natural l-amino acids which could be used by nearby cells for growth and repair processes, and several peptide combinations can inhibit glial scar formation while promoting axonal elongation. Despite these advantages, the technique of self-assembly has the limitation of forming macrosized pores and mechanically unstable 3D structures.^27^

## 3 Cell Therapy: The Most Common Cells Used for Transplantation

Cell therapy is a subtype of regenerative medicine characterized by the introduction of cells into tissues to treat a disease with or without the addition of gene therapy.^36^ Commonly, a particular class of cells, namely “stem cells”, is used for transplantation since they display two essential characteristics: the ability of unlimited self-renewal to produce progeny exactly the same as the originating cell and the ability to give rise to a specialized cell type that becomes part of the healthy organism.^37^ Moreover, stem cells are particularly appealing due to their ability to release several growth factors and immunomodulatory and angiogenic molecules which can further enhance the therapeutic effect (paracrine effect).^38^ Furthermore, cellular differentiation potency plays a key role in stem cell therapy. As a matter of fact, unipotent stem cells have not been excessively used in research due to their ability to create cells with only one lineage differentiation. On the other hand, totipotent, pluripotent, and multipotent cells are frequently chosen. More specifically, totipotent and pluripotent cells have the potential for developing several cellular lineages, while multipotent stem cells can produce a variety of cells limited to a germinal layer or just a specific cell line.^39^ A variety of stem cells exist, and the most common with their key features are summarized in Table 2. *Mesenchymal stem cells* (MSCs) are a subset of nonhematopoietic adult stem cells that originate from the mesoderm which possess self-renewal ability and multilineage differentiation (multipotent cells).^36^ MSCs were first isolated in 1974 by Friedenstein and colleagues^40^ and currently constitute the most promising stem cells in preclinical and clinical research due to their relative ease of access and efficient *in vitro* expansion. Moreover, they are free of ethical concerns and, since they can be used in autologous transplants, are less likely to elicit a significant immune response.^41,42^ MSCs can be collected from different sources such as bone marrow, umbilical cord, amniotic liquid, and adipose tissue leading to possible variable therapeutic results, and other factors, such as the age of a donor or the presence of some disease, affect the therapy efficacy.^43^

**Table 2:** Classification of Stem Cells with Their Most Important Characteristics

stem cell type	characteristics	ref
MSCs	• self-renewal ability	(36, 40−43)
	• multipotent	
	• ease of access	
	• efficient *in vitro* expansion	
	• free of ethical concerns	
	• nonsignificant immune responses	
	• variable therapeutic results depending on the source and the donor	
ESCs	• pluripotent	(42, 44−48)
	• ethical concerns	
	• possible tumor formation during the differentiation	
	• possible immune rejection after transplantation	
	• possible cell heterogeneity	
iPSCs	• pluripotent	(41, 42, 47−50)
	• free of ethical concerns	
	• possible tumor formation during the differentiation	
	• possible immune rejection after transplantation	
	• possible cell heterogeneity	
NSCs	• multipotent	(42, 47, 50−52)
	• efficient *in vitro* expansion	
	• secretion of neurotrophic factors	
	• less potential of forming tumors compared with ESCs	
Schwann cells	• production of growth factors, cell adhesion molecules, and extracellular matrix proteins	(41, 47)
	• myelinating function	
	• support of axonal regeneration	
	• efficacy and safety	

Animal-derived *embryonic stem cells* (ESCs) were successfully cultured for the first time in 1981 by Evans and Kaufman,^44^ while human ESCs (hESCs) were first reported by James Thomson’s group in 1998.^45^ ESCs are an important class of stem cells since they can differentiate into almost all tissues in the human body and are thus labeled as pluripotent due to their ability to produce tissues from all three germ layers (ectoderm, mesoderm, and endoderm) when transplanted.^46^ However, the collection of ESCs (from the inner cell mass of the blastocyst, human oocytes, and human embryos) has raised ethical concerns and the possibility of forming tumors during the differentiation requires precautions in their management.^47^ Moreover, immune rejection after transplantation and heterogeneity of hESC lines have been reported.^48^ Thus, despite the encouraging findings from ESC studies, some concerns remain.^42^ The development of *induced pluripotent stem cells* (iPSCs) by Yamanaka and colleagues^49^ provides a valid alternative to ESCs since iPSCs are characterized by properties similar to ESCs, without particular ethical concerns and are suitable for autologous transplantation. Nevertheless, iPSCs and ESCs share some disadvantages, such as the risk of forming teratomas, transplant reaction,^42^ and cell heterogeneity.^48^ In detail, iPSC technology is based on the derivation of patient-specific and pluripotent cells from adult mouse or human somatic cells by introducing several defined transcription factors^50^ and showed interesting results in preclinical studies.^41^ However, safety issues associated with the manipulation of this type of cell could limit their clinical applicability.^47^ Alternatively, neural stem cells and Swann cells could be used. *Neural stem cells* (NSCs) are multipotent, self-renewing progenitor or stem cells able to differentiate into neurons, oligodendrocytes, and astrocytes, which can be efficiently propagated *in vitro*, are capable of secreting neurotrophic factors, and have less potential to form tumors compared with ESCs.^50^ NSCs are isolated from the subventricular and subgranular zones of the hippocampus of the brain and from a region of the central canal of the spinal cord.^51^

Although several rodent studies have provided prominent results,^41,52^ more mechanistic studies are needed to understand how the environment dictates the differentiation of these cells, and it seems that the source of transplanted NSCs and the methods of isolation and preparation of cells prior to implantation are very critical in cell survival and integration after implantation.^47,50^*Schwann cells* are glial cells with a myelinating function, only present in the peripheral nervous system, where they spontaneously support axonal regeneration after damage.^47^ They offer several properties that could enhance nervous system recovery, such as the production of a variety of growth factors, cell adhesion molecules, and extracellular matrix proteins, and their efficacy and safety have been demonstrated in a variety of preclinical and clinical studies.^41^

## 4 Central Nervous System (CNS) Diseases and Their Treatment with Biomaterials and Cell Therapy Combination

Injury to the CNS can be due to a trauma (e.g., traumatic brain injury, spinal cord injury, stroke), degeneration (e.g., age-related degeneration, Alzheimer’s disease, Parkinson’s disease, multiple sclerosis), or genetic disorder (e.g., Huntington’s disease, retinitis pigmentosa), all leading to cellular degeneration, cellular death, and loss of function.^53^ These pathologies are considered among the most difficult to treat, and the majority still lack an effective and permanent cure because of the inability of the CNS for spontaneous functional regeneration, the complexity of the system, and its numerous protective barriers.^54^ Furthermore, to achieve successful therapeutic treatments, it is necessary to address a variety of challenges that are specific for each injury or disease, which can be broadly defined as “replacing dead neural cells, remodeling the extracellular matrix to a healthy state, and restoring nervous system functionality”.^55^

As previously mentioned, transplant of NSCs can be used as a therapy to heal CNS tissue damage since these cells are able to proliferate and differentiate, leading to repopulation of the damaged tissue.^56^ Many preclinical studies have shown the potential of NSC injection into the injured CNS; however, the transplant result is influenced by the local microenvironment, cell survival and integration remain significant challenges, and it is possible that cells alone do not restore functionality to preinjury baselines.^55−57^ To improve transplantation conditions, biomaterial-based cell therapies can be used. For instance, in the work of Tseng and colleagues,^58^ a self-healing chitosan-based hydrogel was used for NSC transplantation in zebrafish embryos. The encapsulation of NSC spheroids in this hydrogel was an easy and favorable approach, and the cells had a great tendency to differentiate into neuronlike cells *in vitro*. Moreover, animal recovery after the injection of dispersed NSCs without gel was similar to that of the untreated group, while the self-healing hydrogel alone was able to partially rescue the central nervous system (38% recovery rate) *in vivo*. Anyway, NSC addition to the self-healing hydrogel was proven to be the best option, even though it only slightly enhanced the functional recovery to about 43%. A hydrogel’s thermal responsiveness can also be exploited to achieve easy preparation and injection of cell suspensions, and the spontaneous self-assembling at body temperature is useful to tune the hydrogel stiffness to be similar to that of the CNS tissue. An example of this is the diblock copolypeptide hydrogel (DCH) of Zhang and colleagues^59^ consisting of both a hydrophilic part and a hydrophobic part. When used for NSC transplantation in mice, the DCH significantly increased the survival of the cells with respect to the culture media and the grafted NSCs gave rise to new neural cells that distributed throughout the tissue lesions. In addition, natural and synthetic hydrogels can be combined with proteins to improve their cellular entrapment ability. In the work of Addington and colleagues,^60^ for instance, hyaluronic acid has been combined with laminin (HA–Lm) and it was able to increase the transplant retention and migration of neural progenitor/stem cells (NPSCs) with respect to the culture media in rats.

Although these are just a few examples, they confirm the feasibility of biomaterials in cell therapy and show how their use is typically linked to a better transplant outcome. Thus, this review will focus on examples of the use of this combined strategy in preclinical models of the most impacting CNS diseases and peripheral nervous system (PNS) injuries. For what concerns the CNS diseases, spinal cord injury, traumatic brain injury, stroke, Parkinson’s disease, and Alzheimer’s disease will be taken into consideration, while nerve defects, such as sciatic nerve injuries, will be used to describe PNS regeneration therapies.

### 4.1 Spinal Cord Injury

**Figure 2:**
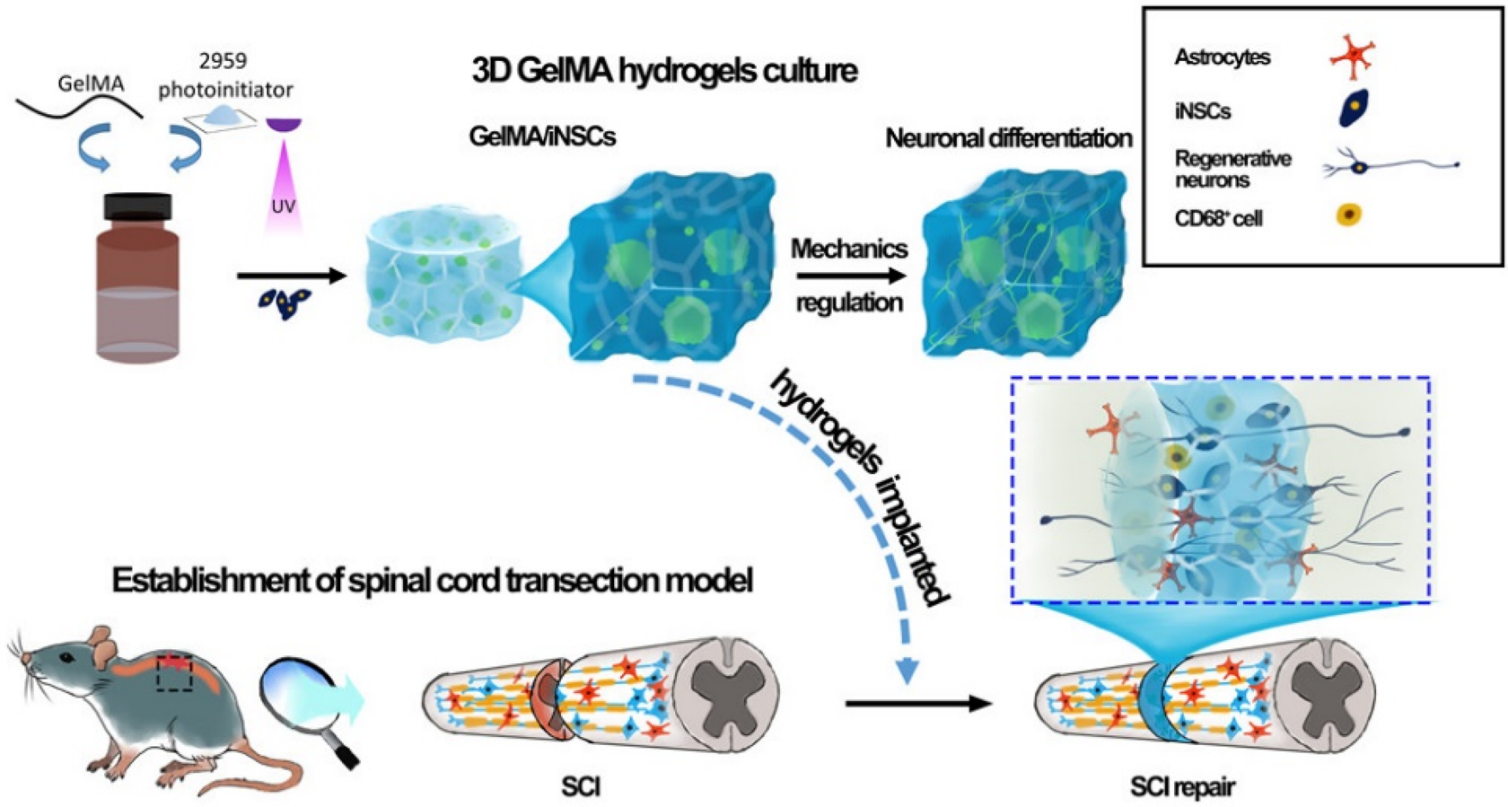
Schematic representation of the hydrogel synthesis and animal experiment. A mixed solution of GelMA and iNSCs cross-linked by a photoinitiator under UV irradiation was developed. After generation of the complete transection mouse SCI model, the scaffold was transplanted into the injury site. MEFs = mouse embryonic fibroblasts; iPSCs = induced pluripotent stem cells; iNSCs = iPSC-derived neural stem cells; NSCs = neural stem cells; RN = regenerative nerve; SCI = spinal cord injury. Reprinted from ref (67). Copyright 2018 American Chemical Society.

Spinal cord injury (SCI) is an acute lesion of the neuronal elements in the spinal canal^61^ representing one of the first causes of disability in the world, with an incidence between 40 and 80 cases per million people yearly.^62^ SCIs are typically due to a traumatic event, such as falls or motor vehicle accidents, and lead to a loss of sensibility and paralysis below the level of injury.^63^ The differences in outcomes are due to different injury levels along the vertebral column and by the lesion’s completeness; clearly, with more severe injuries and older patients recovery is less likely to occur.^64^ Moreover, spastic contractions, skin sensibility loss, autonomic dysreflexia, loss of bladder and bowel control, pain or burning sensation, breathing difficulties, and circulatory problems are common consequences of SCIs.^62^ Nowadays an effective therapy does not exist. Surgical intervention to realign and stabilize the spinal column, and decompression of the spinal cord early after SCI, should help to limit injury extension and improve clinical outcomes.^65^ Furthermore, since the neural tissue is progressively lost after SCI, neuroprotective and neuroregenerative drugs can be administered; however, many pharmacological treatments show limited therapeutic benefits and harmful side effects.^66^ Thus, tissue engineering approaches, such as biomaterial-based cell transplantation directly in the injured site, are promising options. Gelatin methacrylate (GelMa) hydrogels, for instance, can be used as scaffolds in cell therapy approaches (Figure 2) because they share similar characteristics with nerve tissue, as shown in the work of Fan and colleagues.^67^

In detail, GelMa hydrogel with an iNSC photoencapsulated implant was able to significantly enhance functional recovery the decrease inflammation and the lesion cavity area while simultaneously promoting axonal regeneration. The same results have been obtained by another work group^68^ with a serotonin-modified pHEMA hydrogel; however, the gel did not provide ideal long-term support for the continued growth and differentiation of NSCs, probably due to the aggressive SCI environment. Moreover, chondroitin sulfate methacrylate and methacrylamide chitosan (CSMA) based scaffolds can be used for guiding the differentiation of NSCs *in vivo* promoting neurogenesis and functional recovery.^65,66^ ECM-based natural scaffolds have a great potential to be developed for the treatment of SCI, as stated by the Afsartala research group,^71^ who transplanted MSCs encapsulated in either collagen (Col) or fibrin (Fibr) scaffolds, obtaining an increased animal functional recovery in both cases. However, Geissler and colleagues^72^ showed that better *in vivo* results in terms of functional recovery, reduction of the lesion cavity, and transplanted cell differentiation can be achieved with a combination of natural polymers, such as collagen, laminin, and hyaluronic acid (Col–HA–Lam hydrogel), with respect to the singular component scaffolds. To further improve transplant outcomes, growth factors and proteins can be encapsulated within the scaffolds so that a more favorable environment for stem cells is created within SCI sites.^73−76^ Moreover, peptide modification of the hydrogel components or peptide coatings are useful to promote the adhesive growth of transplanted cells.^77−80^ Lastly, Günther and colleagues^81^ were able to physically guide axon orientation in order to increase spinal cord regeneration by transplanting MSCs in alginate-based hydrogels characterized by an anisotropic capillary structure. Analogous results have been obtained by transplanting NSCs with PGA and PLGA–PEG nanofibers.^82,83^ Moreover, Tavakol and colleagues^84^ exploited a thermogel called Matrigel which forms nanofibers at 37 °C mimicking the ECM for the transplant of cells in rodents, obtaining prominent results.

Furthermore, many SAPs have been used for stem cell transplants. The Zweckberger group and the Iwasaki group,^85,86^ for instance, studied the transplant of QL6 peptide scaffold with NPCs. In detail, the SAP scaffold has been injected into the injured site after 24 h, while NPC transplantation has been delayed for 14 days so that the scaffold could ameliorate the hostile injury environment, mitigate components of the secondary injury cascade, and reduce the barriers to neuroregeneration while increasing the number of surviving cells and enhancing their differentiation. Optimal *in vivo* results have been obtained also with nanofibrous scaffolds based on other SAPs such as HYDROSAP, CQIK, RADA4, and RADA16.^87−90^ In addition, some clinical trials with a collagen scaffold called NeuroRegen and MSCs have been reported. Zhao and colleagues^91^ tested the NeuroRegen–MSC implant in eight patients with chronic SCI, demonstrating that it is safe and feasible for clinical therapy. During the 1 year follow-up no adverse events were observed while primary efficacy outcomes, such as expansion of sensation level and motor-evoked potential responsive area, increased finger activity, enhanced trunk stability, defecation sensation, and autonomic neural function recovery, were observed in some patients. The Xiao research group^92^ repeated the trial with two patients, confirming the results and improving the injury status from complete injury (ASIA grade A) to incomplete injury (ASIA grade C).

### 4.2 Traumatic Brain Injury

Traumatic brain injuries (TBIs) can affect people of all ages and are a major cause of death and disability, with an incidence of around 10 million people worldwide. They include penetrating injuries, in which an object breaches the skull and dura, and closed-head injuries, in which the skull and dura remain intact.^93^ TBIs can be categorized into mild, moderate, and severe based on clinical factors, and clearly signs and symptoms vary by severity, ranging from loss of consciousness to coma or even death. Mild TBIs represent the majority of cases; however, moderate and severe injuries can happen, and these are neurosurgical and intensive care concerns.^94^

Therapeutic approaches include pharmacological and surgical strategies which present some limitations. From the pharmacological point of view, fast clearance of drugs represents the principal obstacle leading to a hampered prolonged release, while for surgical procedures there is a need for biocompatible materials that can substitute for physiological tissues and promote recovery.^54^ In this context, scaffolds and cell therapies have been combined. For instance, hyaluronic acid based scaffolds can be used with promising results due to their good injectability, stability, biodegradability, and biocompatibility. In particular, Zhang and colleagues^95^ developed a composite hydrogel scaffold of sodium alginate and hyaluronic acid characterized by a high water content and slow degradation speed exhibiting optimal porosity and rheological properties for MSC loading and differentiation which contribute to the regeneration of endogenous nerve cells in a mild TBI rat model. Moreover, Wang and colleagues^96^ were able to obtain a higher recovery and an accelerated healing process in a rat model by incapsulating in a cross-linked hyaluronic acid hydrogel nerve growth factor (NGF) able to provide a nutritional supply for MSCs while suppressing neuroinflammation and apoptosis. Fibroblast growth factor-2 (FGF-2) can be also chosen to enhance transplant outcomes as shown by Skop and colleagues with a chitosan–fibronectin scaffold.^97^ Easy injection can be obtained using thermoresponsive polymers as well. Polyurethane dispersions, for instance, form gels near 37 °C without any cross-linkers and cell encapsulation is possible before gelation, as shown by Hsieh and colleagues,^98^ who were able to repair the CNS damaged tissue of adult zebrafish with a polyurethane gel containing NSCs. Among the nanofibrous scaffolds, PGA fibers and self-assembling peptides have been used for TBI recovery. For instance, Shin and colleagues^82^ used a PGA scaffold for the transplant of NPCs in mice, showing that the scaffold increased cell engraftment and differentiation, while the combined strategy reduced the lesion cavity volume, increased neovascularization, promoted neurite outgrowth and axonal extension within the lesion site, and facilitated the connection of damaged neural circuits.

**Figure 3:**
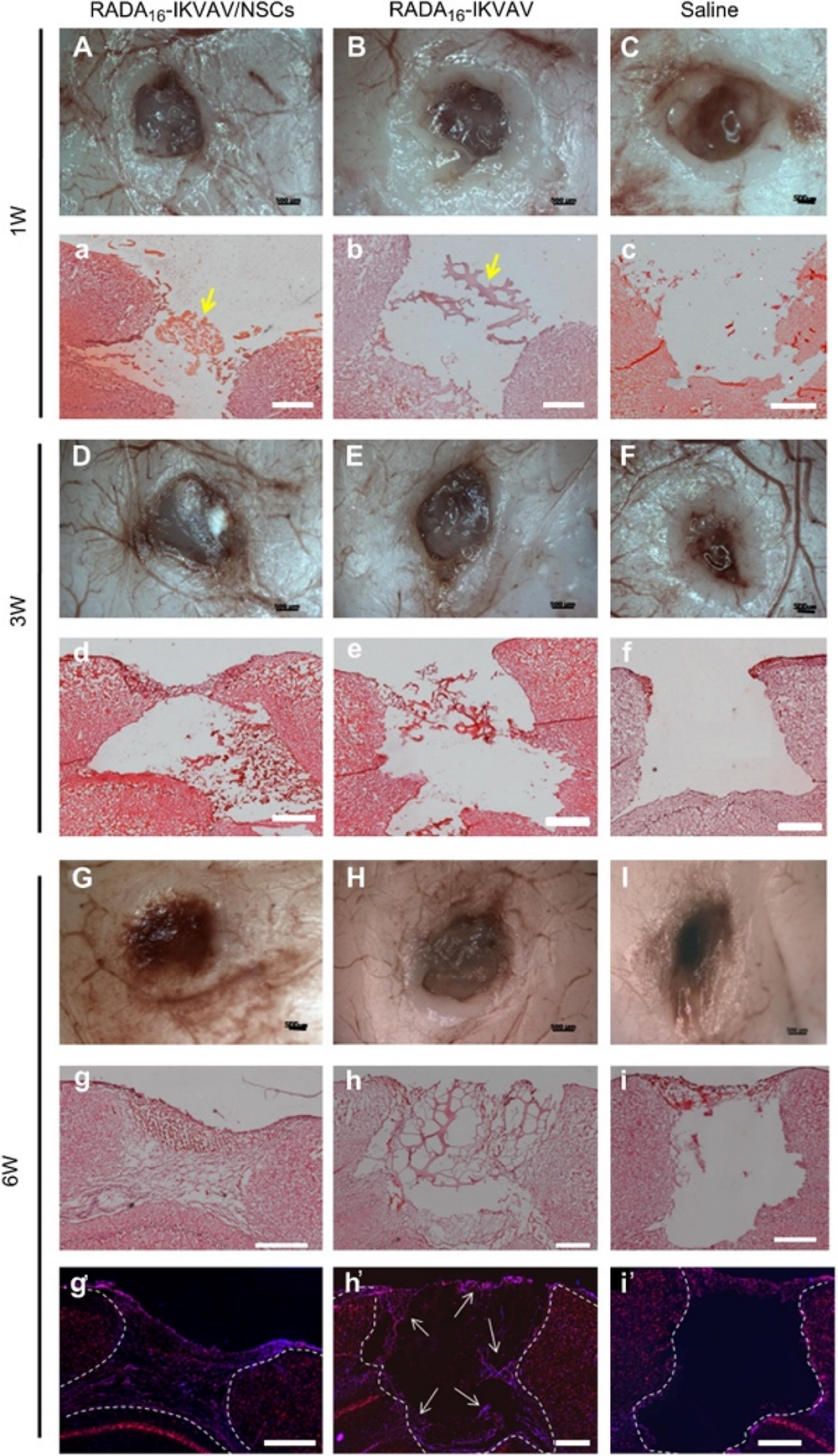
Gross morphological examinations of brain wound defect (A–I) and H/E staining of brain neural tissue in coronary sections (a–i). Neurons were labeled with red fluorescent Nissl stain, and the nuclei were counterstained with blue fluorescent DAPI in coronal sections 6 weeks after surgery (g′–i′). The yellow arrows point out the remaining SAP hydrogels in the injured cavities. The white dashed lines outline the wound margin to distinguish the area of original host tissue and neoregenerated neural tissue. Scale bar = 200 μm. Reproduced with permission from ref (99). Copyright 2012 Elsevier.

On the other hand, scaffolds obtained from the SAP RADA16 are interesting since some moieties can be linked to the RADA16 C-terminal (Figure 3). As shown in the works of Cheng and colleagues^99^ and Shi and colleagues^100^ where laminin-derived and brain derived growth factor (BDGF) peptide derived moieties were respectively added to RADA16, the peptide modification led to enhanced cell encapsulation, proliferation, and differentiation in mice TBI models with moderate-size lesion cavity healing.

### 4.3 Stroke

Stroke is the second highest cause of death globally and a leading cause of disability, with an increasing incidence in developing countries.^101^ It defines all conditions in which the cerebral blood flow does not provide sufficient oxygen and/or glucose to the brain for an excess of 24 h.^102^ It is broadly classified into hemorrhagic stroke, which includes intracerebral and subarachnoid hemorrhage, and ischemic stroke, which is caused by the occlusion of a vascular structure within the brain, spinal cord, or retina and represents 71% of all stroke cases.^103^ Following stroke, a brain injury develops from a complex series of pathological events such as depolarization, inflammation, and excitotoxicity which dramatically compromise the stability of the blood–brain barrier (BBB) and activate the release of free radicals and proteases which deepen and extend the injury leading to cell death.^104^ The early recognition of symptoms and the rapidity of medical intervention influence the clinical evolution of each patient. Reperfusion strategies to reestablish the blood flow can be used for recovery after stroke. These are divided into pharmacological approaches such as intravenous thrombolysis and surgical procedures such as endovascular thrombectomy. However, a minority of stroke patients can really get benefits from these treatments due to the narrow time window for the drug administration and the risks of complications.^103^ Thus, stem cell therapy with biomaterials employment constitutes a promising approach to stimulate functional recovery after stroke. Due to its favorable properties, hyaluronic acid based scaffolds have been used to treat rodent strokes. As shown by Moshayedi and colleagues,^105^ the mechanical, biochemical, and biological properties of hyaluronic acid based scaffolds can be optimized to minimize the reactions of brain tissue after implantation. Moreover, by adhesive peptide motifs and growth factor encapsulation, it is possible to promote the *in vivo* survival of iPSCs and NPCs while guiding cell differentiation to glial and neuronal states.^106^

Physical blends of hyaluronic acid and methylcellulose (HAMC) have been used by Ballios and colleagues^107^ and Payne and colleagues^108^ to transplant NPCs in mice stroke models, obtaining better results with respect to conventional buffered saline vehicles in terms of cell penetration and distribution and behavioral recovery. These results underline how important is the biomaterial composition for cells’ fate since hyaluronic acid promotes cell survival, but methylcellulose is fundamental to promoting a uniform cellular distribution. Nanofibrous scaffolds have been used as well. For instance, Fernández-García and colleagues^109^ used a silk fibroin self-assembling hydrogel to transplant MSCs, obtaining a longer period of cell engraftment, a progressive and significant recovery, and a reduced extent of brain damage in animals receiving the scaffold with respect to the ones receiving buffered saline solution. On the other hand, Somaa and colleagues^110^ fabricated a scaffold using SAPs able to not only structurally and functionally support neural grafts but also promote cell graft differentiation and integration. Moreover, the combination of this scaffold with ESCs led to a reduction in the host tissue atrophy, improving mice motor functions over a period of 9 months. Lastly, Bliss and colleagues^111^ demonstrated how the electrical preconditioning of NPCs using a conductive scaffold of polypyrrole and the transplantation of this system 7 days after stroke lead to an enhancement of the recovery in mice.

### 4.4 Parkinson’s and Alzheimer’s Diseases

Parkinson’s disease (PD) is the most common neurodegenerative movement disorder that affects 0.3% of the population in industrialized countries, and incidence rates are estimated to range between 8 and 18 new cases per 100 000 people yearly.^112^ Although it is an age-related disease, with incidence and prevalence increasing steadily with age, almost 25% of affected individuals are younger than 65 years and 5–10% are younger than 50 years (e.g., young-onset Parkinson’s disease).^113^

PD is due to a neuronal loss in the substantia nigra which causes striatal dopamine deficiency, leading to a movement disorder characterized by classical Parkinsonian motor symptoms and numerous nonmotor symptoms with a continuous slow progression of the disease over time and accumulating disability for affected individuals.^114^ Moreover, symptoms can vary among people and the earliest stages of the disease can be difficult to recognize, due to the long delay (average 10 years) that typically separates the person’s first noticeable symptom from the timing of diagnosis.^113^ Substituting striatal dopamine loss via the systemic administration of the dopamine precursor amino acid l-DOPA has remained the gold standard for Parkinson’s disease treatment. However, its use is complicated by the evolution of motor complications and the discontinuous drug delivery due to the short half-life of l-DOPA and the variability in its gastrointestinal absorption and blood–brain barrier transport.^115^ Thus, cell therapies with the idea of transplanting dopamine-producing cells, derived from hESCs or from iPSCs, to selectively restore dopamine loss can be used. Adil and colleagues,^116^ for instance, transplanted human ESC derived midbrain dopaminergic neurons in rodents through an RGD peptide/heparin modified hyaluronic acid scaffold enriched with growth factors. This strategy led to an enhanced cell replacement therapy with respect to cell injection with a clear alleviation of PD symptoms. In addition, Struzyna and colleagues^117^ transplanted dopaminergic neurons in rats by exploiting agarose and ECM hydrogel microcolumns. The dopaminergic neurons were able to release dopamine and synapse with striatal neurons in the brain, but the real advantage of this transplant method is the microcolumn structure of the biomaterial which permits the simultaneous replacement of neurons in the substantia nigra and the reconstruction of their axonal tracts to the striatum. Midbrain dopamine progenitors, on the other hand, have been transplanted by Wang and colleagues^118^ with an injectable composite scaffold of PLLA nanofibers embedded within a thermoresponsive xyloglucan hydrogel. Also in this case, no immune responses were provoked in Parkinsonian mice and the reinnervation of the striatum was enhanced by the introduction of glial derived neurotrophic factor (GDNF) within the scaffold.

Moreover, SAP scaffolds can be used for midbrain dopamine progenitor transplant, as shown by Rodriguez and colleagues.^119^ In this case, the peptide was chosen to promote neural differentiation and neurite elongation, while GDNF was added to promote the survival of the transplanted neurons. Better recovery results could be observed in mice when the scaffold and cell combination strategy was used. Lastly, not only dopaminergic grafts but also more common stem cells such as MSCs and NSCs can be used for PD treatment by directing their neuronal differentiation with an appropriate scaffold. To do so, Das and colleagues^120^ proposed a scaffold composed by self-assembling amyloid proteins to promote MSC survival and differentiation without the need for growth factors, while Nakaji-Hirabayashi and colleagues^121^ used a collagen hydrogel incorporating integrin-binding proteins for NSC transplantation in the striatum.

Alzheimer’s disease (AD) is a neurodegenerative disorder characterized by gradually progressive cognitive and functional deficits as well as behavioral changes.^122^ Cognitive symptoms of AD include deficits in short-term memory and impairment in expressive speech, visuospatial processing, and executive functions.^123^ Although many data show that AD pathology starts developing in the brain in midlife, the first clinical symptoms usually occur after the age of 65 years.^122^ In addition, although age and genetics are the most important risk factors, AD development is multifactorial since type 2 diabetes, hypertension, smoking, sedentary lifestyle, obesity, and head injury contribute to the disease evolution.^124^ Unfortunately, disease-modifying agents, i.e., those proven to alter the underlying disease pathology or disease course, are not yet available, so supportive care is the most common treatment for AD, which needs to be tailored to the individual patient and their specific circumstances and adapted as the disease progresses.^125^ Most studies show that the lifestyle strategies including physical activity, mental challenges, energy restriction, socialization, and good sleep act as preventive factors in AD and pharmacological intervention helps with both cognitive and noncognitive symptoms, although there is no evidence that one drug is more efficacious than another.^124,125^

Even cell therapies are not commonly used for AD treatment, although biomaterial employment can lead to cognitive rescue with restoration of learning/memory function and synaptic function as shown by Cui and colleagues.^126^ Their data clearly demonstrate how a designed SAP scaffold can maximize the therapeutic benefits of NSC transplantation for AD by improving the survival and differentiation of transplanted cells and promoting neuroprotection, antineuroinflammatory, and paracrine action underlining that biomaterial-based stem cell therapy can be a reliable strategy to relieve AD symptoms.

## 5 Beyond CNS: Peripheral Nervous System Regeneration

The peripheral nervous system (PNS) refers to the nerves connecting the central nervous system to the entire human body, typically divided into somatic and autonomic nerves with distinct functions. The somatic system transmits sensory information for the CNS, while the autonomic one controls automatic functions (e.g., heart beating, blood pressure).^127^ Opposite to the CNS, which is enclosed by the vertebrae and the skull, the PNS is not protected by bones and therefore is more susceptible to trauma and peripheral nerve injuries (PNIs).^128^ As a matter of fact, PNIs can happen through many other events such as infections, autoimmune disorders, alcohol, toxins, and even medications, and it is considered one of the leading causes of permanent dysfunctionality and morbidity, due to CNS disconnection from the limbs.^129^ The PNS has more capacity for neuroregeneration with respect to the CNS due to the favorable presence of Schwann cells;^130^ however, reliable treatments that allow for complete recovery are rare and injuries larger than 1 cm have limited solutions for functional recovery.^33^ Therefore, reconstruction surgery is required, and autologous, allogeneic, and xenogeneic nerve grafts can be chosen. Allograft and xenograft usage is hampered by limited resources and the risk of immunological rejection, so autografting is considered the gold standard technique.

However, it is still characterized by some limitations, such as the second surgery required to obtain donor nerves, possible morbidities and secondary deformities at the donor site, and mismatches between the damaged and donor nerves. Thus, neural tissue engineering is promising to guide the regeneration of peripheral nerve tissue and effectively avoid immune rejection, inflammation, and disease transmission.^131^ A common technique for PNS regeneration is the employment of bio artificial nerve substitutes composed of a conduit filled with a hydrogel scaffold containing cells. For instance, NeuraGen collagen conduits filled with a sterile fibrin–agarose hydrogel and MSCs transplanted in a rat sciatic nerve model can avoid the additional donor site morbidity associated with autografts while producing an effective nerve regeneration characterized by properly oriented axons and a partial sensory recovery.^132,133^ However, the composition of the conduits is extremely important for the success of the therapy and a matrix giving good *in vitro* results does not guarantee the same *in vivo*.^134^ In addition, cell type influences the results, as shown by Gonzalez-Perez and colleagues,^135^ who indicated Schwann cell grafts as the best alternative to autografts. Bulk hydrogels can be used as well. The Salehi research group,^136^ for instance, showed how an alginate/chitosan hydrogel can be used for the regeneration of a rat sciatic nerve defect underlining how the addition of MSCs was able to significantly enhance the process with respect to the control group and the hydrogel alone. Moreover, for the treatment of acute PNS injuries, Demyanenko and colleagues^137^ performed a preclinical study by transplanting a collagen-based hydrogel containing stem cell culture media derived extracellular vesicles in a rat sciatic nerve model, obtaining interesting results from the regeneration point of view. SAP scaffolds are optimal even for PNI treatment since they provide a permissive environment for NSC/NPC transplant, allow cell differentiation without the need for factors, and favor native Schwann cell recruitment enhancing the regeneration process as demonstrating by Sun and colleagues^138^ for three nerve injury models.

**Figure 4:**
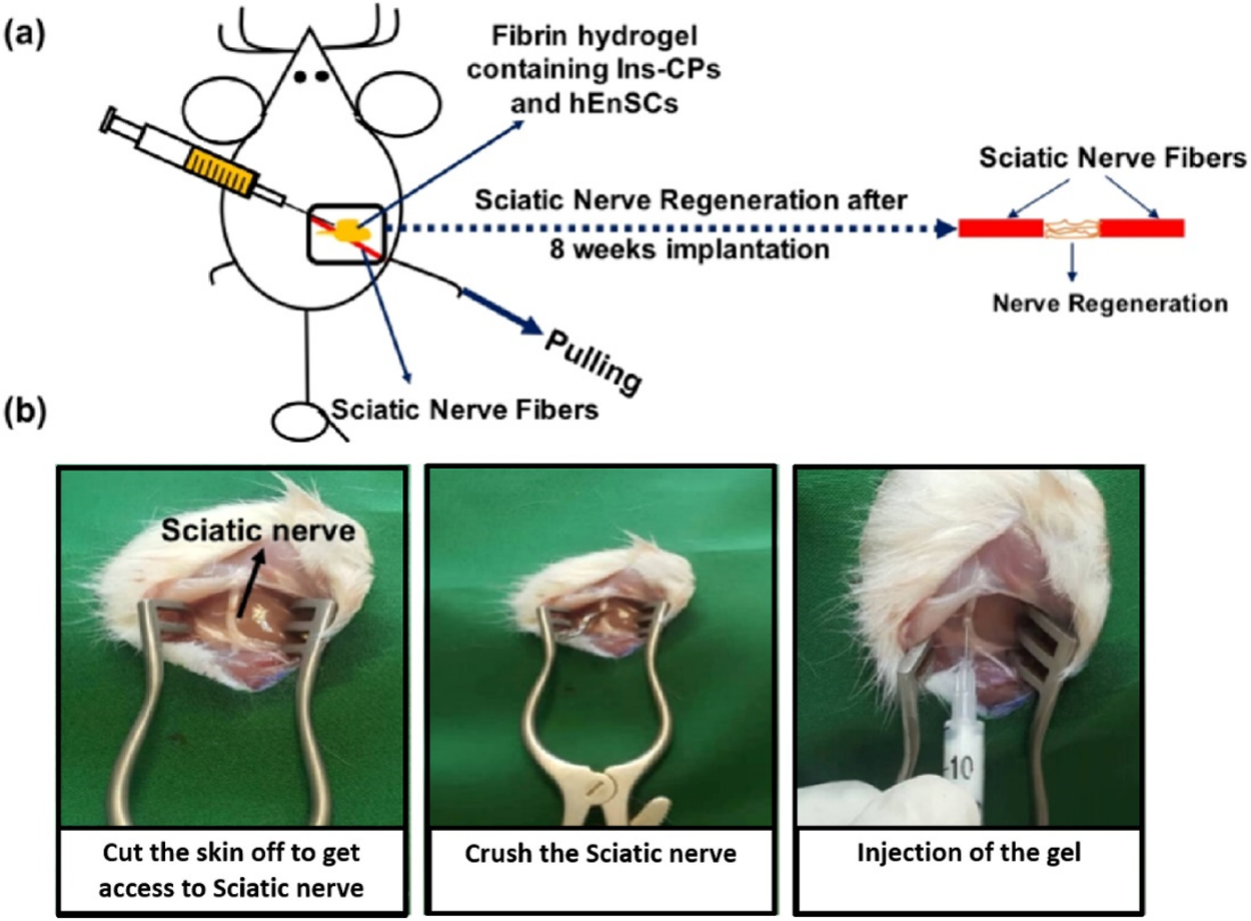
(a) Schematic overview of the surgical injection of fibrin hydrogel in order to heal sciatic nerve damage in rats. The sciatic nerve of the rat was obtained by making a skin incision, and a 4-mm-long sciatic nerve crush injury was created by exerting a constant force. Prepared fibrin gel was injected at the site of crush injury to regenerate sciatic nerve injury overtimes. (b) Steps of surgical injection of fibrin hydrogel containing Ins-CPs and hEnSCs to bridge a 4 mm sciatic nerve defect in rats. Reproduced with permission from ref (139). Copyright 2023 Elsevier.

To conclude, a novel approach of using a composite scaffold has been shown by the Mobarakeh research group.^139^ In detail, a fibrin gel was combined with chitosan nanoparticles containing insulin, which was slowly released promoting transplanted stem cell proliferation and enhancing the survival of mature neurons, vascularization, and neurological regeneration *in vivo* (Figure 4).

## 6 Methodology

We performed our research through the PubMed interface and Google Scholar to identify preclinical studies combining biomaterials and stem cells for nervous tissue regeneration. We used the Boolean operator “AND” to merge keywords, resulting in several search strings different for any neurological disorder or injury. For further comprehension, here is reported a search string example: hydrogel[Title/Abstract]) AND cell[Title/Abstract] AND sci[Title/Abstract]. The search was restricted to preclinical and clinical trials, the English language, and the year of publication, 2013–2023. Furthermore, the exclusion criteria were the following: (1) only *in vitro* evaluation of the treatment; (2) treatments including biomaterial only; (3) treatments including cells only; (4) treatment of a different, but correlated disease (i.e., caused by a CNS injury).

## 7 Conclusions

In conclusion, this review highlights the significant advances in the development and application of hydrogel and nanofiber scaffolds in neural tissue engineering. As briefly summarized in Table 3, the evidence presented underscores their potential in addressing critical challenges in the regeneration of nervous tissues across various conditions, including SCI, TBI, stroke, PD, AD, and PNI. While these biomaterials have demonstrated promising results in enhancing neural regeneration and functional recovery, the field is still evolving. Future research should focus on exploring the mechanistic pathways of these scaffolds, optimizing their properties for specific neural applications, and addressing translational challenges for clinical applications. Additionally, further investigations into the long-term effects and scalability of these biomaterials are crucial for their practical application in regenerative medicine. Moreover, as underlined by the presented studies, the biomaterial conjunction with stem cells is fundamental for better outcomes owing to the cellular ability of secrete neurotrophic factors and support axonal regeneration. As the field progresses, this combined strategy holds the promise of revolutionizing the treatment of neurological disorders and injuries, offering new hope for recovery and rehabilitation.

**Table 3:** Schematic Summary of the Preclinical Trials Presented in This Review

biomaterial	stem cells	additional factors	outcome	ref
SCI				
GelMA hydrogel	iNSCs	–	• functional recovery promotion	(67)
			• cavity area reduction	
			• reduction of inflammation	
			• axonal regeneration promotion	
pHEMA hydrogel	NSCs	serotonin	• acceleration of cellular differentiation *in vitro*/*in vivo*	(68)
			• initial reduction of tissue atrophy and glial scar formation	
			• nonideal long-term support for cellular growth and differentiation	
CSMA hydrogel	NSCs	–	• controlled differentiation of NSCs *in vitro*/*in vivo*	(69)
			• cavity area reduction	
			• neurogenesis and functional recovery promotion	
methacrylamide scaffold contained in a chitosan channel (nerve conduit)	NPCs	interferon-g (IFN-g), platelet derived growth factor-AA (PDGF-AA), or bone morphogenic protein-2 (BMP-2) growth factor	• only *in vitro* results: NSPC differentiation is maintained at functionally significant levels for 28 days	(70)
			• growth factor immobilization induced the majority of cells to differentiate into desired cell types as compared with adsorbed growth factor treatments and controls by day 28 *in vivo*	
collagen and fibrin hydrogels	MSCs	–	• functional recovery promotion	(71)
			• no significant differences between collagen and fibrin hydrogels in terms of functional recovery	
Col–HA–Lam hydrogel	NPCs	–	• lesion size reduction	(72)
			• functional recovery promotion	
			• longer-term response examination is needed	
hyaluronan and methyl cellulose (HAMC) hydrogel	NSCs/NPCs	recombinant rPDGF-A	• functional recovery promotion	(73)
			• cavity area reduction	
			• improvement of graft survival	
HAMC–RGD peptide hydrogel	hiPSCs	PDGF-A	• early survival and integration of cell promotion	(74)
			• cell differentiation promotion and attenuation of teratoma formation (when cells were transplanted in the hydrogel)	
			• teratoma formation when cells were transplanted in media	
fibrin hydrogel	ESCs	neurotrophin-3 (NT3) and PDGF-AA or NT3 and GDNF	• improvement of cell survival with a delayed transplant	(75)
			• cellular differentiation promotion	
			• the presence of growth factors did not appear to influence survival or proliferation of transplanted cells	
MC hydrogel	hiPSCs	chondroitinase ABC (chABC)	• lesion cavity reduction	(76)
			• no motor function improvement	
			• chABC favored neuronal survival and differentiation	
gellan gum (GG)–GRGDS peptide hydrogel	adipose stromal stem cells (hASCs) and murine olfactory ensheathing cells (OECs)	–	• GG–GRGDS hydrogel is suitable for cellular culture	(77)
			• neurite/axonal outgrowth promotion *in vitro*	
			• significant motor and histological improvements *in vivo*	
HA–PPFLMLLKGSTR peptide hydrogel	MSCs	–	• improved cellular survival and adhesive growth *in vitro*	(78)
			• scaffold and MSCs are found to function in synergy	
			• injured spinal cord tissue restoration and motor functions improvement	
poly(acrylic acid)/agarose/PEG (AC PEG) and AC PEG–RGD peptide hydrogels with 3D ECM deposition	hMSCs	–	• immunomodulation of the pro-inflammatory environment in a SCI mouse model promoting a proregenerative environment in situ	(79)
poly(sebacoyl diglyceride) (PSeD)–IKVAVS peptide scaffold	NSCs	–	• reduction of direct stimulation to spinal cord tissue by PSeD elastomer	(80)
			• reduction of immune response of spinal cord tissue and of scar tissue formation	
			• increase of locomotor recovery	
			• IKVAVS peptide creates a bioactive interface to support NSC growth and differentiation	
alginate-base anisotropic capillaries	MSCs	–	• higher number of axons expressing BDNF in the hydrogel compared to control cells	(81)
			• nonsignificant differences in the number of regenerating axons increasing the channel diameter	
			• the anisotropic structure can physically guide regenerating axons	
PGA fibers	NPCs	–	• lesion volume reduction	(82)
			• survival, engraftment, and differentiation of grafted cell promotion	
			• neovascularization increase	
			• glial scar formation inhibition	
			• neurite outgrowth and axonal extension within the lesion site promotion	
			• significant improvement of motosensory function	
			• neuropathic pain attenuation	
PLGA–PEG fibers with gelatin sponge coating	iNSCs	–	• survival, engraftment, and differentiation of grafted cell promotion	(83)
			• functional recovery promotion	
Matrigel (nanofibrous scaffold)	human endometrial-derived stromal cells (hEnSCs)	–	• differentiation of encapsulated hEnSCs toward neuronlike cells after 14 days posttreatment	(84)
			• significantly higher cellular viability in Matrigel compared with 2D cell culture	
			• damaged tissue reconstruction	
			• decrease of cavity size, degree of necrosis, and number of glial and inflammatory cells around the injury site	
			• significant improvement in motor function of the injured animals	
QL6 peptide scaffold (nanofibrous)	NPCs	–	• QL6 SAP injection into the SCI site 24 h after trauma, NPC transplantation 14 days after trauma	(85, 86)
			• QL6 scaffold shaped the hostile posttraumatic microenvironment improving transplant conditions (NPCs surviving)	
			• astrogliosis and tissue-scarring reduction	
			• significant recovery of forelimb neural function	
HYDROSAP peptide scaffold (nanofibrous)	hNSCs	–	• formation of an entangled network of mature and functional neural phenotypes with 3D cell culture model	(87)
			• astrogliosis and immune response reduction	
			• scaffolds with predifferentiated hNSCs showed higher percentages of neuronal markers, better hNSC engraftment, and improved behavioral recovery with respect to hNSC-derived progenitors	
CQIK–RADA4 peptide scaffold (nanofibrous)	hEnSCs	–	• CQIK induces hEnSC transformation to neurallike cell after 10 days postincubation *in vitro*	(88)
			• significant motor recovery, neurogenesis, and antiastrogliosis potential	
RADA16 peptide scaffold (nanofibrous)	human cerebral microvascular endothelial cells (HCMEC/D3)	–	• cellular growth, proliferation, and migration within the scaffold	(89)
			• vascularization and axon growth support	
			• glial scar, inflammation, and immune response minimization	
RADA16–RGD peptide scaffold (nanofibrous)	MSCs	–	• MSC and neuron survival improvement	(90)
			• inflammatory reaction inhibition	
			• functional behaviors promotion	
NeuroRegen (collagen) scaffold	MSCs	–	• no adverse events observed during 1 year of follow-up	(91, 92)
			• recovery of sensory and motor functions	
			• recovery of interrupted neural conduction	
TBI				
sodium alginate (SA) and HA hydrogel	MSCs	–	• high cellular viability and proliferation within the scaffold *in vitro*	(95)
			• cell protection from the injury environment	
			• cellular survival improvement *in vivo*	
			• endogenous nerve cell regeneration	
HA hydrogel	MSCs	NGF	• hydrogel implantation provides a positive nutrition supply for cell survival and proliferation	(96)
			• significant promotion of functional recovery of motor, learning, and memory abilities	
			• acceleration of the healing process of damaged brain tissues	
			• neuroinflammation and apoptosis suppression	
chitosan/heparin-modified fibronectin hydrogel	radial glial cells (RGCs)	FGF-2	• the hydrogel can be used as a cellular and growth factor delivery vehicle to promote the regeneration of nervous tissue	(97)
			• more detailed *in vivo* studies are required to assess cellular survival and differentiation as well as detailing the extent of anatomical and functional recovery	
polyurethane gel	NSCs	–	• favorable proliferation and differentiation of cells within the scaffold	(98)
			• repair of damaged CNS and functional recovery promotion *in vivo*	
PGA fibers	NPCs	–	• lesion volume reduction	(82)
			• survival, engraftment, and differentiation of grafted cell promotion	
			• neovascularization increase	
			• neurite outgrowth and axonal extension within the lesion site promotion	
			• connection of damaged neural circuits improvement	
RADA16–IKVAV peptide scaffold (nanofibrous)	NSCs	–	• NSC proliferation and differentiation promotion	(99)
			• *in situ* support and bridging of damaged brain wounds	
RADA16–RGIDKRHWNSQ peptide scaffold (nanofibrous)	MSCs	–	• BDNF-derived peptide (RGIDKRHWNSQ) introduced to promote neurotrophy, cell proliferation, neuronal differentiation, and neurite outgrowth	(100)
			• brain cavity and surrounding reactive gliosis reduction	
			• large cavity repair is not promoted	
Stroke				
heparin-modified HA–RGD, YIGSR, IKVAV peptide hydrogel	iPSCs and NPCs	BMP-4 and BDNF growth factors	• *in vivo* promotion of cell survival and differentiation after transplantation into the stroke core	(105)
HA–RGD peptide hydrogel	iPSCs and NPCs	–	• differentiation of the neural progenitor cells to neuroblasts promotion	(106)
			• stem cell viability 1 week posttransplantation nonpromotion	
HAMC hydrogel	NSCs		• cell survival improvement (due to HA)	(107)
			• better cellular depth of penetration and distribution (due to MC)	
			• significant behavioral recovery in the animal model of stroke	
HAMC hydrogel	cortically specified neuroepithelial progenitor cells (cNEPs)	–	• greater and faster functional repair with undifferentiated progenitor cells	(108)
			• great tissue damage, acute cell death during the transplantation process and no functional repair with late differentiated cell injection	
silk fibroin self-assembling hydrogel	MSCs	–	• longer period cell engraftment within the scaffold	(109)
			• cortical damage reduction and progressive and significant recovery in stroke mice	
DDIKVAV peptide scaffold (nanofibrous)	hESCs	–	• structural and functional support of neural grafts in a stroke model	(110)
			• cell graft differentiation and integration promotion	
			• host tissue atrophy reduction resulting in improved motor function over a period of 9 months	
polypyrrole scaffold	hNPCs	–	• functional outcome improvement with NPCs electrically preconditioning	(111)
PD				
HA–RGD–heparin hydrogel	hESC-derived midbrain dopaminergic neuron	–	• cell replacement enhancement	(116)
			• alleviation of disease symptoms	
agarose hydrogel microcolumns with ECM coating	dopaminergic neurons with long axonal tracts	–	• dopamine is released by the transplanted neurons	(117)
			• simultaneous replace of dopaminergic neurons in the substantia nigra and physical reconstruction of their long axonal tracts to the striatum	
PLLA short nanofibers embedded within a thermoresponsive xyloglucan hydrogel	ventral midbrain (VM) dopamine progenitors	GDNF	• no deleterious impact on the host immune response *in vivo*	(118)
			• survival and integration of grafted neurons enhancement	
			• reinnervation of the striatum	
minimalist *N*-fluorenylmethyloxycarbonyl (Fmoc)–DIKVAV peptide scaffolds (nanofibrous)	VM cell grafts	GDNF	• DIKVAV introduced to promote neural differentiation and neurite elongation	(119)
			• GDNF introduced to promote survival and neurite extension of neuron grafts	
			• sustained release of GDNF up to 172 h after gel loading	
			• improvement of graft survival *in vivo*	
self-assembling amyloid proteins hydrogel (nanofibrous)	hMSCs	–	• promotion of MSCs differentiation *in vitro*/*in vivo* toward a neuronal lineage without the addition of growth factors	(120)
			• nontoxic hydrogel	
			• no excessive immune response	
			• optimal cellular containment at injury site and improved survival *in vivo*	
collagen hydrogel	NSCs	collagen-binding LG3 (CLG3) and histidine tagged LP (HLP), an integrin-binding protein complex	• NSC viability improvement in the early stage after transplantation into the striatum due to integrin ligation and microglial infiltration suppression	(121)
AD				
RADA16–YIGSR peptide scaffold (nanofibrous)	NSCs	–	• cellular migration, survival, and neuronal differentiation improvement	(126)
			• decrease of the neuronal apoptosis and synaptic loss	
			• the scaffold provided a trophic support to modulate inflammation and facilitate neuroprotection, neurogenesis, and antineuroinflammatory	
PNI				
NeuraGen (collagen) guides filled with fibrin–agarose hydrogels (FAH)	MSCs	–	• superior clinical, electrophysiological, and histological results at 12 weeks after repair with hydrogel alone, better outcomes with hydrogel/MSCs	(132, 133)
			• lower percentage of self-amputations	
			• partial sensory and motor function recovery	
			• active peripheral nerve regeneration process with newly formed peripheral nerve fascicles and remyelination	
			• regeneration process more abundant in autograft group	
			• important weight and volume loss	
			• additional donor site morbidity	
			• some signs of atrophy and fibrosis	
NVR-Gel (hydrogel of high MW HA and laminin)	SCs	GDNF or FGF-2 expressed by SCs	• genetic modification of SCs obtaining a cellular neurotrophic factor delivery system	(134)
			• optimal hydrogel matrix *in vitro* but not *in vivo*	
			• conversion of the NVR-Gel into a solid state as a forward step	
chitosan conduits filled with cellular collagen type I scaffolds enriched with either fibronectin or laminin	MSCs and Schwann cells	–	• marked improvement of regeneration and functional recovery	(135)
			• highest values of regenerated nerves area using SCs (nonsignificant differences among all groups)	
alginate/chitosan hydrogel	MSCs	–	• the hydrogel can provide a suitable substrate for cell survival *in vitro*/*in vivo*	(136)
			• enhance regeneration compared to control group and hydrogel without cells	
collagen type I and III hydrogel	extracellular vesicles (EVs) isolated from hMSC cultured media	–	• reduction of muscle atrophy	(137)
			• functional recovery of innervated muscle enhancement	
			• EV-induced neuroprotective mechanisms	
RADA16–RGD–IKVAV peptide scaffold (nanofibrous)	NPCs and NSCs	–	• good survival of NPCs/NSCs when fully embedded in the 3D environment of the nanofiber hydrogel	(138)
			• NPC differentiation into neurons and astrocytes without adding extra soluble growth factors within the scaffold *in vitro*	
			• more permissive environment for nerve regeneration with RADA16–RGD–IKVAV with respect to RADA16 alone	
fibrin gel with chitosan nanoparticles (NPs)	hEnSCs	insulin (in chitosan NPs)	• insulin slow release (possible with chitosan NPs) to improve matrix regeneration and neovascularization	(139)
			• hEnSC proliferation promotion within a certain concentration range of insulin *in vitro*	
			• significant motor function and sensory recovery improvement while forming regenerative nerve fibers accompanied by new blood vessels	

## References

[ref1] Melchels F. P. W.; Domingos M. A. N.; Klein T. J.; Malda J.; Bartolo P. J.; Hutmacher D. W. Additive Manufacturing of Tissues and Organs. Prog. Polym. Sci. 2012, 37 (8), 1079–1104. 10.1016/j.progpolymsci.2011.11.007.

[ref2] Rana D.; Arulkumar S.; Vishwakarma A.; Ramalingam M. Considerations on Designing Scaffold for Tissue Engineering. In Stem Cell Biology and Tissue Engineering in Dental Sciences; Elsevier: 2015; pp 133–148. 10.1016/B978-0-12-397157-9.00012-6.

[ref3] Luo Y.; Engelmayr G.; Auguste D. T.; da Silva Ferreira L.; Karp J. M.; Saigal R.; Langer R. 3D Scaffolds. In Principles of Tissue Engineering, 4th ed.; Elsevier: 2014; pp 475–494. 10.1016/B978-0-12-398358-9.00024-0.

[ref4] Dvir T.; Timko B. P.; Kohane D. S.; Langer R. Nanotechnological Strategies for Engineering Complex Tissues. Nat. Nanotechnol. 2011, 6 (1), 13–22. 10.1038/nnano.2010.246. 21151110 PMC4059057

[ref5] Lee K. Y.; Mooney D. J. Hydrogels for Tissue Engineering. Chem. Rev. 2001, 101 (7), 1869–1880. 10.1021/cr000108x. 11710233

[ref6] Perale G.; Rossi F.; Sundstrom E.; Bacchiega S.; Masi M.; Forloni G.; Veglianese P. Hydrogels in Spinal Cord Injury Repair Strategies. ACS Chem. Neurosci. 2011, 2 (7), 336–345. 10.1021/cn200030w. 22816020 PMC3369745

[ref7] Catoira M. C.; Fusaro L.; Di Francesco D.; Ramella M.; Boccafoschi F. Overview of Natural Hydrogels for Regenerative Medicine Applications. J. Mater. Sci. Mater. Med. 2019, 30 (10), 115 10.1007/s10856-019-6318-7. 31599365 PMC6787111

[ref8] Casalini T.; Perale G. From Microscale to Macroscale: Nine Orders of Magnitude for a Comprehensive Modeling of Hydrogels for Controlled Drug Delivery. Gels 2019, 5 (2), 28 10.3390/gels5020028. 31096685 PMC6631542

[ref9] Richbourg N. R.; Wancura M.; Gilchrist A. E.; Toubbeh S.; Harley B. A. C.; Cosgriff-Hernandez E.; Peppas N. A. Precise Control of Synthetic Hydrogel Network Structure via Linear, Independent Synthesis-Swelling Relationships. Sci. Adv. 2021, 7 (7), eabe3245 10.1126/sciadv.abe3245. 33579714 PMC7880590

[ref10] Ghane N.; Beigi M.-H.; Labbaf S.; Nasr-Esfahani M.-H.; Kiani A. Design of Hydrogel-Based Scaffolds for the Treatment of Spinal Cord Injuries. J. Mater. Chem. B 2020, 8 (47), 10712–10738. 10.1039/D0TB01842B. 33155614

[ref11] Madhusudanan P.; Raju G.; Shankarappa S. Hydrogel Systems and Their Role in Neural Tissue Engineering. J. R Soc. Interface 2020, 17 (162), 20190505 10.1098/rsif.2019.0505. 31910776 PMC7014813

[ref12] Shao Y.; Jia H.; Cao T.; Liu D. Supramolecular Hydrogels Based on DNA Self-Assembly. Acc. Chem. Res. 2017, 50 (4), 659–668. 10.1021/acs.accounts.6b00524. 28299927

[ref13] Liu J.; Zheng H.; Poh P. S. P.; Machens H. G.; Schilling A. F. Hydrogels for Engineering of Perfusable Vascular Networks. Int. J. Mol. Sci. 2015, 16, 15997–16016. 10.3390/ijms160715997. 26184185 PMC4519935

[ref14] Madduma-Bandarage U. S. K.; Madihally S. v. Synthetic Hydrogels: Synthesis, Novel Trends, and Applications. J. Appl. Polym. Sci. 2021, 138 (19), 50376 10.1002/app.50376.

[ref15] Mohamed M. A.; Fallahi A.; El-Sokkary A. M. A.; Salehi S.; Akl M. A.; Jafari A.; Tamayol A.; Fenniri H.; Khademhosseini A.; Andreadis S. T.; Cheng C. Stimuli-Responsive Hydrogels for Manipulation of Cell Microenvironment: From Chemistry to Biofabrication Technology. Prog. Polym. Sci. 2019, 98, 101147 10.1016/j.progpolymsci.2019.101147. 36467305 PMC9718481

[ref16] Casolaro M.; Casolaro I.; Lamponi S. Stimuli-Responsive Hydrogels for Controlled Pilocarpine Ocular Delivery. Eur. J. Pharm. Biopharm 2012, 80 (3), 553–561. 10.1016/j.ejpb.2011.11.013. 22138000

[ref17] Buwalda S. J.; Vermonden T.; Hennink W. E. Hydrogels for Therapeutic Delivery: Current Developments and Future Directions. Biomacromolecules 2017, 18 (2), 316–330. 10.1021/acs.biomac.6b01604. 28027640

[ref18] Sood N.; Bhardwaj A.; Mehta S.; Mehta A. Stimuli-Responsive Hydrogels in Drug Delivery and Tissue Engineering. Drug Delivery 2016, 23 (3), 748–770. 10.3109/10717544.2014.940091. 25045782

[ref19] Sosnik A.; Sefton M. v. Semi-Synthetic Collagen/Poloxamine Matrices for Tissue Engineering. Biomaterials 2005, 26 (35), 7425–7435. 10.1016/j.biomaterials.2005.05.086. 16023714

[ref20] Palmese L. L.; Thapa R. K.; Sullivan M. O.; Kiick K. L. Hybrid Hydrogels for Biomedical Applications. Curr. Opin. Chem. Eng. 2019, 24, 143–157. 10.1016/j.coche.2019.02.010. 31844607 PMC6914224

[ref21] Xie X.; Chen Y.; Wang X.; Xu X.; Shen Y.; Khan A. ur R.; Aldalbahi A.; Fetz A. E.; Bowlin G. L.; El-Newehy M.; Mo X. Electrospinning Nanofiber Scaffolds for Soft and Hard Tissue Regeneration. J. Mater. Sci. Technol. 2020, 59, 243–261. 10.1016/j.jmst.2020.04.037.

[ref22] Ma B.; Xie J.; Jiang J.; Shuler F. D.; Bartlett D. E. Rational Design of Nanofiber Scaffolds for Orthopedic Tissue Repair and Regeneration. Nanomedicine 2013, 8 (9), 1459–1481. 10.2217/nnm.13.132. 23987110 PMC3875778

[ref23] Garg T.; Rath G.; Goyal A. K. Biomaterials-Based Nanofiber Scaffold: Targeted and Controlled Carrier for Cell and Drug Delivery. J. Drug Target 2015, 23 (3), 202–221. 10.3109/1061186X.2014.992899. 25539071

[ref24] Yang F.; Xu C. Y.; Kotaki M.; Wang S.; Ramakrishna S. Characterization of Neural Stem Cells on Electrospun Poly(L-Lactic Acid) Nanofibrous Scaffold. J. Biomater Sci. Polym. Ed 2004, 15 (12), 1483–1497. 10.1163/1568562042459733. 15696794

[ref25] Chen Y.; Shafiq M.; Liu M.; Morsi Y.; Mo X. Advanced Fabrication for Electrospun Three-Dimensional Nanofiber Aerogels and Scaffolds. Bioact Mater. 2020, 5 (4), 963–979. 10.1016/j.bioactmat.2020.06.023. 32671291 PMC7334396

[ref26] Zong X.; Ran S.; Fang D.; Hsiao B. S.; Chu B. Control of Structure, Morphology and Property in Electrospun Poly(Glycolide-Co-Lactide) Non-Woven Membranes via Post-Draw Treatments. Polymer (Guildf) 2003, 44 (17), 4959–4967. 10.1016/S0032-3861(03)00464-6.

[ref27] Cao H.; Liu T.; Chew S. Y. The Application of Nanofibrous Scaffolds in Neural Tissue Engineering. Adv. Drug Deliv Rev. 2009, 61 (12), 1055–1064. 10.1016/j.addr.2009.07.009. 19643156

[ref28] Qian J.; Lin Z.; Liu Y.; Wang Z.; Lin Y.; Gong C.; Ruan R.; Zhang J.; Yang H. Functionalization Strategies of Electrospun Nanofibrous Scaffolds for Nerve Tissue Engineering. Smart Mater. Med. 2021, 2, 260–279. 10.1016/j.smaim.2021.07.006.

[ref29] Whitesides G. M.; Mathias J. P.; Seto C. T. Molecular Self-Assembly and Nanochemistry: A Chemical Strategy for the Synthesis of Nanostructures. Science (1979) 1991, 254 (5036), 1312–1319. 10.1126/science.1962191. 1962191

[ref30] Zhang S.; Lockshin C.; Herbert A.; Winter E.; Rich A. Zuotin, a Putative Z-DNA Binding Protein in Saccharomyces Cerevisiae. EMBO J. 1992, 11 (10), 3787–3796. 10.1002/j.1460-2075.1992.tb05464.x. 1396572 PMC556839

[ref31] Zhang S.; Holmes T.; Lockshin C.; Rich A. Spontaneous Assembly of a Self-Complementary Oligopeptide to Form a Stable Macroscopic Membrane. Proc. Natl. Acad. Sci. U. S. A. 1993, 90 (8), 3334–3338. 10.1073/pnas.90.8.3334. 7682699 PMC46294

[ref32] Zhang S.; Holmes T. C.; DiPersio C. M.; Hynes R. O.; Su X.; Rich A. Self-Complementary Oligopeptide Matrices Support Mammalian Cell Attachment. Biomaterials 1995, 16 (18), 1385–1393. 10.1016/0142-9612(95)96874-Y. 8590765

[ref33] Peressotti S.; Koehl G. E.; Goding J. A.; Green R. A. Self-Assembling Hydrogel Structures for Neural Tissue Repair. ACS Biomater Sci. Eng. 2021, 7 (9), 4136–4163. 10.1021/acsbiomaterials.1c00030. 33780230 PMC8441975

[ref34] Gelain F.; Luo Z.; Zhang S. Self-Assembling Peptide EAK16 and RADA16 Nanofiber Scaffold Hydrogel. Chem. Rev. 2020, 120 (24), 13434–13460. 10.1021/acs.chemrev.0c00690. 33216525

[ref35] Maude S.; Ingham E.; Aggeli A. Biomimetic Self-Assembling Peptides as Scaffolds for Soft Tissue Engineering. Nanomedicine 2013, 8 (5), 823–847. 10.2217/nnm.13.65. 23656267

[ref36] Wei X.; Yang X.; Han Z.; Qu F.; Shao L.; Shi Y. Mesenchymal Stem Cells: A New Trend for Cell Therapy. Acta Pharmacol Sin 2013, 34 (6), 747–754. 10.1038/aps.2013.50. 23736003 PMC4002895

[ref37] Biehl J. K.; Russell B. Introduction to Stem Cell Therapy. Journal of Cardiovascular Nursing 2009, 24 (2), 98–103. 10.1097/JCN.0b013e318197a6a5. 19242274 PMC4104807

[ref38] Veneruso V.; Rossi F.; Villella A.; Bena A.; Forloni G.; Veglianese P. Stem Cell Paracrine Effect and Delivery Strategies for Spinal Cord Injury Regeneration. J. Controlled Release 2019, 300, 141–153. 10.1016/j.jconrel.2019.02.038. 30851286

[ref39] Rajabzadeh N.; Fathi E.; Farahzadi R. Stem Cell-Based Regenerative Medicine. Stem Cell Investig 2019, 6, 19 10.21037/sci.2019.06.04. PMC6691074 31463312

[ref40] Friedenstein A. J.; Deriglasova U. F.; Kulagina N. N.; Panasuk A. F.; Rudakowa S. F.; Luriá E. A.; Ruadkow I. A. Precursors for Fibroblasts in Different Populations of Hematopoietic Cells as Detected by the in Vitro Colony Assay Method. Exp. Hematol. 1974, 2 (2), 83–92. 4455512

[ref41] Vismara I.; Papa S.; Rossi F.; Forloni G.; Veglianese P. Current Options for Cell Therapy in Spinal Cord Injury. Trends Mol. Med. 2017, 23 (9), 831–849. 10.1016/j.molmed.2017.07.005. 28811172

[ref42] Digma L. A.; Upadhyayula P. S.; Martin J. R.; Ciacci J. D. Stem Cells and Chronic Spinal Cord Injury: Overview. In Diagnosis and Treatment of Spinal Cord Injury; Elsevier: 2022; pp 397–409. 10.1016/B978-0-12-822498-4.00031-2.

[ref43] Lukomska B.; Stanaszek L.; Zuba-Surma E.; Legosz P.; Sarzynska S.; Drela K. Challenges and Controversies in Human Mesenchymal Stem Cell Therapy. Stem Cells Int. 2019, 2019, 1–10. 10.1155/2019/9628536. PMC6481040 31093291

[ref44] Evans M. J.; Kaufman M. H. Establishment in Culture of Pluripotential Cells from Mouse Embryos. Nature 1981, 292 (5819), 154–156. 10.1038/292154a0. 7242681

[ref45] Thomson J. A.; Itskovitz-Eldor J.; Shapiro S. S.; Waknitz M. A.; Swiergiel J. J.; Marshall V. S.; Jones J. M. Embryonic Stem Cell Lines Derived from Human Blastocysts. Science (1979) 1998, 282 (5391), 1145–1147. 10.1126/science.282.5391.1145. 9804556

[ref46] Ding D.-C.; Chang Y.-H.; Shyu W.-C.; Lin S.-Z. Human Umbilical Cord Mesenchymal Stem Cells: A New Era for Stem Cell Therapy. Cell Transplant 2015, 24 (3), 339–347. 10.3727/096368915X686841. 25622293

[ref47] Gomes E. D.; Rocha L. A.; Assunção-Silva R. C.; Lima R.; Silva N. A.; Salgado A. J. Cell Therapies for Spinal Cord Injury Regeneration. In Spinal Cord Injury (SCI) Repair Strategies; Elsevier: 2020; pp 157–186. 10.1016/B978-0-08-102807-0.00009-0.

[ref48] Yamanaka S. Pluripotent Stem Cell-Based Cell Therapy—Promise and Challenges. Cell Stem Cell 2020, 27 (4), 523–531. 10.1016/j.stem.2020.09.014. 33007237

[ref49] Yamanaka S.; Takahashi K. Induction of Pluripotent Stem Cells from Mouse Fibroblast Cultures. Tanpakushitsu Kakusan Koso 2006, 51 (15), 2346–2351. 17154061

[ref50] Ronaghi M.; Erceg S.; Moreno-Manzano V.; Stojkovic M. Challenges of Stem Cell Therapy for Spinal Cord Injury: Human Embryonic Stem Cells, Endogenous Neural Stem Cells, or Induced Pluripotent Stem Cells?. Stem Cells 2010, 28 (1), 93–99. 10.1002/stem.253. 19904738

[ref51] Grochowski C.; Radzikowska E.; Maciejewski R. Neural Stem Cell Therapy—Brief Review. Clin Neurol Neurosurg 2018, 173, 8–14. 10.1016/j.clineuro.2018.07.013. 30053745

[ref52] Papa S.; Vismara I.; Veglianese P. Paracrine Effects for Spinal Cord Injury Regeneration. In Spinal Cord Injury (SCI) Repair Strategies; Elsevier: 2020; pp 203–221. 10.1016/B978-0-08-102807-0.00011-9.

[ref53] Pakulska M. M.; Ballios B. G.; Shoichet M. S. Injectable Hydrogels for Central Nervous System Therapy. Biomedical Materials 2012, 7 (2), 024101 10.1088/1748-6041/7/2/024101. 22456684

[ref54] Djoudi A.; Molina-Peña R.; Ferreira N.; Ottonelli I.; Tosi G.; Garcion E.; Boury F. Hyaluronic Acid Scaffolds for Loco-Regional Therapy in Nervous System Related Disorders. Int. J. Mol. Sci. 2022, 23 (20), 12174 10.3390/ijms232012174. 36293030 PMC9602826

[ref55] Meco E.; Lampe K. J. Microscale Architecture in Biomaterial Scaffolds for Spatial Control of Neural Cell Behavior. Front Mater. 2018, 5, 2 10.3389/fmats.2018.00002.

[ref56] Tejeda G.; Ciciriello A. J.; Dumont C. M. Biomaterial Strategies to Bolster Neural Stem Cell-Mediated Repair of the Central Nervous System. Cells Tissues Organs 2023, 211 (6), 655–669. 10.1159/000515351. 34120118

[ref57] Ho M. T.; Teal C. J.; Shoichet M. S. A Hyaluronan/Methylcellulose-Based Hydrogel for Local Cell and Biomolecule Delivery to the Central Nervous System. Brain Res. Bull. 2019, 148, 46–54. 10.1016/j.brainresbull.2019.03.005. 30898580

[ref58] Tseng T.-C.; Tao L.; Hsieh F.-Y.; Wei Y.; Chiu I.-M.; Hsu S. An Injectable, Self-Healing Hydrogel to Repair the Central Nervous System. Adv. Mater. 2015, 27 (23), 3518–3524. 10.1002/adma.201500762. 25953204

[ref59] Zhang S.; Burda J. E.; Anderson M. A.; Zhao Z.; Ao Y.; Cheng Y.; Sun Y.; Deming T. J.; Sofroniew M. V. Thermoresponsive Copolypeptide Hydrogel Vehicles for Central Nervous System Cell Delivery. ACS Biomater Sci. Eng. 2015, 1 (8), 705–717. 10.1021/acsbiomaterials.5b00153. 27547820 PMC4991036

[ref60] Addington C. P.; Dharmawaj S.; Heffernan J. M.; Sirianni R. W.; Stabenfeldt S. E. Hyaluronic Acid-Laminin Hydrogels Increase Neural Stem Cell Transplant Retention and Migratory Response to SDF-1α. Matrix Biology 2017, 60–61, 206–216. 10.1016/j.matbio.2016.09.007. PMC5357205 27645115

[ref61] Chen Y.; Tang Y.; Vogel L.; DeVivo M. Causes of Spinal Cord Injury. Top Spinal Cord Inj Rehabil 2013, 19 (1), 1–8. 10.1310/sci1901-1. 23678280 PMC3584795

[ref62] Papa S.; Mauri E.; Rossi F.; Perale G.; Veglianese P. Introduction to Spinal Cord Injury as Clinical Pathology. In Spinal Cord Injury (SCI) Repair Strategies; Elsevier: 2020; pp 1–12. 10.1016/B978-0-08-102807-0.00001-6.

[ref63] Flórez-Jiménez S.; Bourassa-Moreau C9.; Mac-Thiong J.-M.; Maurais G. Biomechanics and Patterns of Spine Injuries Associated with Spinal Cord Injury. In Diagnosis and Treatment of Spinal Cord Injury; Elsevier: 2022; pp 15–25. 10.1016/B978-0-12-822498-4.00002-6.

[ref64] Yohann A.; Purcell L. N.; Charles A. Traumatic Spinal Cord Injury and Outcomes in Low-Resource Settings. In Diagnosis and Treatment of Spinal Cord Injury; Elsevier: 2022; pp 3–14. 10.1016/B978-0-12-822498-4.00001-4.

[ref65] Ahuja C. S.; Wilson J. R.; Nori S.; Kotter M. R. N.; Druschel C.; Curt A.; Fehlings M. G. Traumatic Spinal Cord Injury. Nat. Rev. Dis Primers 2017, 3, 17018 10.1038/nrdp.2017.18. 28447605

[ref66] Witiw C. D.; Fehlings M. G. Acute Spinal Cord Injury. J. Spinal Disord Tech 2015, 28 (6), 202–210. 10.1097/BSD.0000000000000287. 26098670

[ref67] Fan L.; Liu C.; Chen X.; Zou Y.; Zhou Z.; Lin C.; Tan G.; Zhou L.; Ning C.; Wang Q. Directing Induced Pluripotent Stem Cell Derived Neural Stem Cell Fate with a Three-Dimensional Biomimetic Hydrogel for Spinal Cord Injury Repair. ACS Appl. Mater. Interfaces 2018, 10 (21), 17742–17755. 10.1021/acsami.8b05293. 29733569

[ref68] Růžička J.; Romanyuk N.; Hejčl A.; Vetrík M.; Hrubý M.; Cocks G.; Cihlár J.; Přádný M.; Price J.; Syková E.; Jendelová P. Treating Spinal Cord Injury in Rats with a Combination of Human Fetal Neural Stem Cells and Hydrogels Modified with Serotonin. Acta Neurobiol Exp (Wars) 2013, 73 (1), 102–115. 10.55782/ane-2013-1925. 23595287

[ref69] Liu C.; Fan L.; Xing J.; Wang Q.; Lin C.; Liu C.; Deng X.; Ning C.; Zhou L.; Rong L.; Liu B. Inhibition of Astrocytic Differentiation of Transplanted Neural Stem Cells by Chondroitin Sulfate Methacrylate Hydrogels for the Repair of Injured Spinal Cord. Biomater Sci. 2019, 7 (5), 1995–2008. 10.1039/C8BM01363B. 30839020

[ref70] Li H.; Koenig A. M.; Sloan P.; Leipzig N. D. In Vivo Assessment of Guided Neural Stem Cell Differentiation in Growth Factor Immobilized Chitosan-Based Hydrogel Scaffolds. Biomaterials 2014, 35 (33), 9049–9057. 10.1016/j.biomaterials.2014.07.038. 25112933

[ref71] Afsartala Z.; Hadjighassem M.; Shirian S.; Ebrahimi-Barough S.; Gholami L.; Fahad Hussain M.; Yaghoobi M.; Ai J. Comparison of the Regenerative Effect of Adipose Tissue Mesenchymal Stem Cell Encapsulated into Two Hydrogel Scaffolds on Spinal Cord Injury. Arch Neurosci 2022, 9 (1), e119170 10.5812/ans.119170.

[ref72] Geissler S. A.; Sabin A. L.; Besser R. R.; Gooden O. M.; Shirk B. D.; Nguyen Q. M.; Khaing Z. Z.; Schmidt C. E. Biomimetic Hydrogels Direct Spinal Progenitor Cell Differentiation and Promote Functional Recovery after Spinal Cord Injury. J. Neural Eng. 2018, 15 (2), 025004 10.1088/1741-2552/aaa55c. 29303112 PMC5988207

[ref73] Mothe A. J.; Tam R. Y.; Zahir T.; Tator C. H.; Shoichet M. S. Repair of the Injured Spinal Cord by Transplantation of Neural Stem Cells in a Hyaluronan-Based Hydrogel. Biomaterials 2013, 34 (15), 3775–3783. 10.1016/j.biomaterials.2013.02.002. 23465486

[ref74] Führmann T.; Tam R. Y.; Ballarin B.; Coles B.; Elliott Donaghue I.; van der Kooy D.; Nagy A.; Tator C. H.; Morshead C. M.; Shoichet M. S. Injectable Hydrogel Promotes Early Survival of Induced Pluripotent Stem Cell-Derived Oligodendrocytes and Attenuates Longterm Teratoma Formation in a Spinal Cord Injury Model. Biomaterials 2016, 83, 23–36. 10.1016/j.biomaterials.2015.12.032. 26773663

[ref75] McCreedy D. A.; Wilems T. S.; Xu H.; Butts J. C.; Brown C. R.; Smith A. W.; Sakiyama-Elbert S. E. Survival, Differentiation, and Migration of High-Purity Mouse Embryonic Stem Cell-Derived Progenitor Motor Neurons in Fibrin Scaffolds after Sub-Acute Spinal Cord Injury. Biomater Sci. 2014, 2 (11), 1672–1682. 10.1039/C4BM00106K. 25346848 PMC4206060

[ref76] Führmann T.; Anandakumaran P. N.; Payne S. L.; Pakulska M. M.; Varga B. V.; Nagy A.; Tator C.; Shoichet M. S. Combined Delivery of Chondroitinase ABC and Human Induced Pluripotent Stem Cell-Derived Neuroepithelial Cells Promote Tissue Repair in an Animal Model of Spinal Cord Injury. Biomed. Mater. 2018, 13 (2), 024103 10.1088/1748-605X/aa96dc. 29083317

[ref77] Gomes E. D.; Mendes S. S.; Leite-Almeida H.; Gimble J. M.; Tam R. Y.; Shoichet M. S.; Sousa N.; Silva N. A.; Salgado A. J. Combination of a Peptide-Modified Gellan Gum Hydrogel with Cell Therapy in a Lumbar Spinal Cord Injury Animal Model. Biomaterials 2016, 105, 38–51. 10.1016/j.biomaterials.2016.07.019. 27505621

[ref78] Li L.-M.; Han M.; Jiang X.-C.; Yin X.-Z.; Chen F.; Zhang T.-Y.; Ren H.; Zhang J.-W.; Hou T.-J.; Chen Z.; Ou-Yang H.-W.; Tabata Y.; Shen Y.-Q.; Gao J.-Q. Peptide-Tethered Hydrogel Scaffold Promotes Recovery from Spinal Cord Transection via Synergism with Mesenchymal Stem Cells. ACS Appl. Mater. Interfaces 2017, 9 (4), 3330–3342. 10.1021/acsami.6b12829. 28058831

[ref79] Caron I.; Rossi F.; Papa S.; Aloe R.; Sculco M.; Mauri E.; Sacchetti A.; Erba E.; Panini N.; Parazzi V.; Barilani M.; Forloni G.; Perale G.; Lazzari L.; Veglianese P. A New Three Dimensional Biomimetic Hydrogel to Deliver Factors Secreted by Human Mesenchymal Stem Cells in Spinal Cord Injury. Biomaterials 2016, 75, 135–147. 10.1016/j.biomaterials.2015.10.024. 26497428

[ref80] Gong Z.; Lei D.; Wang C.; Yu C.; Xia K.; Shu J.; Ying L.; Du J.; Wang J.; Huang X.; Ni L.; Wang C.; Lin J.; Li F.; You Z.; Liang C. Bioactive Elastic Scaffolds Loaded with Neural Stem Cells Promote Rapid Spinal Cord Regeneration. ACS Biomater Sci. Eng. 2020, 6 (11), 6331–6343. 10.1021/acsbiomaterials.0c01057. 33449647

[ref81] Günther M. I.; Weidner N.; Müller R.; Blesch A. Cell-Seeded Alginate Hydrogel Scaffolds Promote Directed Linear Axonal Regeneration in the Injured Rat Spinal Cord. Acta Biomater 2015, 27, 140–150. 10.1016/j.actbio.2015.09.001. 26348141

[ref82] Shin J. E.; Jung K.; Kim M.; Hwang K.; Lee H.; Kim I.-S.; Lee B. H.; Lee I.-S.; Park K. I. Brain and Spinal Cord Injury Repair by Implantation of Human Neural Progenitor Cells Seeded onto Polymer Scaffolds. Exp Mol. Med. 2018, 50 (4), 1–18. 10.1038/s12276-018-0054-9. PMC5938022 29674624

[ref83] Liu C.; Huang Y.; Pang M.; Yang Y.; Li S.; Liu L.; Shu T.; Zhou W.; Wang X.; Rong L.; Liu B. Tissue-Engineered Regeneration of Completely Transected Spinal Cord Using Induced Neural Stem Cells and Gelatin-Electrospun Poly (Lactide-Co-Glycolide)/Polyethylene Glycol Scaffolds. PLoS One 2015, 10 (3), e0117709 10.1371/journal.pone.0117709. 25803031 PMC4372351

[ref84] Tavakol S.; Aligholi H.; Gorji A.; Eshaghabadi A.; Hoveizi E.; Tavakol B.; Rezayat S. M.; Ai J. Thermogel Nanofiber Induces Human Endometrial-Derived Stromal Cells to Neural Differentiation: *In Vitro* and *in Vivo* Studies in Rat. J. Biomed. Mater. Res. A 2014, 102 (12), 4590–4597. 10.1002/jbm.a.35117. 24532561

[ref85] Zweckberger K.; Ahuja C. S.; Liu Y.; Wang J.; Fehlings M. G. Self-Assembling Peptides Optimize the Post-Traumatic Milieu and Synergistically Enhance the Effects of Neural Stem Cell Therapy after Cervical Spinal Cord Injury. Acta Biomater 2016, 42, 77–89. 10.1016/j.actbio.2016.06.016. 27296842

[ref86] Iwasaki M.; Wilcox J. T.; Nishimura Y.; Zweckberger K.; Suzuki H.; Wang J.; Liu Y.; Karadimas S. K.; Fehlings M. G. Synergistic Effects of Self-Assembling Peptide and Neural Stem/Progenitor Cells to Promote Tissue Repair and Forelimb Functional Recovery in Cervical Spinal Cord Injury. Biomaterials 2014, 35 (9), 2617–2629. 10.1016/j.biomaterials.2013.12.019. 24406216

[ref87] Marchini A.; Raspa A.; Pugliese R.; El Malek M. A.; Pastori V.; Lecchi M.; Vescovi A. L.; Gelain F. Multifunctionalized Hydrogels Foster HNSC Maturation in 3D Cultures and Neural Regeneration in Spinal Cord Injuries. Proc. Natl. Acad. Sci. U. S. A. 2019, 116 (15), 7483–7492. 10.1073/pnas.1818392116. 30923117 PMC6462084

[ref88] Tavakol S.; Saber R.; Hoveizi E.; Tavakol B.; Aligholi H.; Ai J.; Rezayat S. M. Self-Assembling Peptide Nanofiber Containing Long Motif of Laminin Induces Neural Differentiation, Tubulin Polymerization, and Neurogenesis: In Vitro, Ex Vivo, and In Vivo Studies. Mol. Neurobiol 2016, 53 (8), 5288–5299. 10.1007/s12035-015-9448-z. 26427854

[ref89] Tran K. A.; Partyka P. P.; Jin Y.; Bouyer J.; Fischer I.; Galie P. A. Vascularization of Self-Assembled Peptide Scaffolds for Spinal Cord Injury Repair. Acta Biomater 2020, 104, 76–84. 10.1016/j.actbio.2019.12.033. 31904559

[ref90] Li J.; Ji Z.; Wang Y.; Li T.; Luo J.; Li J.; Shi X.; Li L.; He L.; Wu W. Human Adipose-Derived Stem Cells Combined with Nano-Hydrogel Promote Functional Recovery after Spinal Cord Injury in Rats. Biology (Basel) 2022, 11 (5), 781 10.3390/biology11050781. 35625508 PMC9138297

[ref91] Zhao Y.; Tang F.; Xiao Z.; Han G.; Wang N.; Yin N.; Chen B.; Jiang X.; Yun C.; Han W.; Zhao C.; Cheng S.; Zhang S.; Dai J. Clinical Study of Neuroregen Scaffold Combined with Human Mesenchymal Stem Cells for the Repair of Chronic Complete Spinal Cord Injury. Cell Transplant 2017, 26 (5), 891–900. 10.3727/096368917X695038. 28185615 PMC5657723

[ref92] Xiao Z.; Tang F.; Zhao Y.; Han G.; Yin N.; Li X.; Chen B.; Han S.; Jiang X.; Yun C.; Zhao C.; Cheng S.; Zhang S.; Dai J. Significant Improvement of Acute Complete Spinal Cord Injury Patients Diagnosed by a Combined Criteria Implanted with NeuroRegen Scaffolds and Mesenchymal Stem Cells. Cell Transplant 2018, 27 (6), 907–915. 10.1177/0963689718766279. 29871514 PMC6050906

[ref93] Blennow K.; Brody D. L.; Kochanek P. M.; Levin H.; McKee A.; Ribbers G. M.; Yaffe K.; Zetterberg H. Traumatic Brain Injuries. Nat. Rev. Dis Primers 2016, 2 (1), 16084 10.1038/nrdp.2016.84. 27853132

[ref94] Faul M.; Coronado V. Epidemiology of Traumatic Brain Injury. Handbook of Clinical Neurology 2015, 127, 3–13. 10.1016/B978-0-444-52892-6.00001-5. 25702206

[ref95] Zhang K.; Shi Z.; Zhou J.; Xing Q.; Ma S.; Li Q.; Zhang Y.; Yao M.; Wang X.; Li Q.; Li J.; Guan F. Potential Application of an Injectable Hydrogel Scaffold Loaded with Mesenchymal Stem Cells for Treating Traumatic Brain Injury. J. Mater. Chem. B 2018, 6 (19), 2982–2992. 10.1039/C7TB03213G. 32254333

[ref96] Wang L.; Zhang D.; Ren Y.; Guo S.; Li J.; Ma S.; Yao M.; Guan F. Injectable Hyaluronic Acid Hydrogel Loaded with BMSC and NGF for Traumatic Brain Injury Treatment. Mater. Today Bio 2022, 13, 100201 10.1016/j.mtbio.2021.100201. PMC8733324 35024600

[ref97] Skop N. B.; Calderon F.; Cho C. H.; Gandhi C. D.; Levison S. W. Optimizing a Multifunctional Microsphere Scaffold to Improve Neural Precursor Cell Transplantation for Traumatic Brain Injury Repair. J. Tissue Eng. Regen. Med. 2016, 10 (10), E419–E432. 10.1002/term.1832. 27730762

[ref98] Hsieh F.-Y.; Lin H.-H.; Hsu S. 3D Bioprinting of Neural Stem Cell-Laden Thermoresponsive Biodegradable Polyurethane Hydrogel and Potential in Central Nervous System Repair. Biomaterials 2015, 71, 48–57. 10.1016/j.biomaterials.2015.08.028. 26318816

[ref99] Cheng T.-Y.; Chen M.-H.; Chang W.-H.; Huang M.-Y.; Wang T.-W. Neural Stem Cells Encapsulated in a Functionalized Self-Assembling Peptide Hydrogel for Brain Tissue Engineering. Biomaterials 2013, 34 (8), 2005–2016. 10.1016/j.biomaterials.2012.11.043. 23237515

[ref100] Shi W.; Huang C. J.; Xu X. D.; Jin G. H.; Huang R. Q.; Huang J. F.; Chen Y. N.; Ju S. Q.; Wang Y.; Shi Y. W.; Qin J. B.; Zhang Y. Q.; Liu Q. Q.; Wang X. B.; Zhang X. H.; Chen J. Transplantation of RADA16-BDNF Peptide Scaffold with Human Umbilical Cord Mesenchymal Stem Cells Forced with CXCR4 and Activated Astrocytes for Repair of Traumatic Brain Injury. Acta Biomater 2016, 45, 247–261. 10.1016/j.actbio.2016.09.001. 27592818

[ref101] Katan M.; Luft A. Global Burden of Stroke. Semin Neurol 2018, 38 (02), 208–211. 10.1055/s-0038-1649503. 29791947

[ref102] Cooke M. J.; Vulic K.; Shoichet M. S. Design of Biomaterials to Enhance Stem Cell Survival When Transplanted into the Damaged Central Nervous System. Soft Matter 2010, 6 (20), 4988 10.1039/c0sm00448k.

[ref103] Campbell B. C. V.; De Silva D. A.; Macleod M. R.; Coutts S. B.; Schwamm L. H.; Davis S. M.; Donnan G. A. Ischaemic Stroke. Nat. Rev. Dis Primers 2019, 5 (1), 70 10.1038/s41572-019-0118-8. 31601801

[ref104] Nih L. R.; Carmichael S. T.; Segura T. Hydrogels for Brain Repair after Stroke: An Emerging Treatment Option. Curr. Opin Biotechnol 2016, 40, 155–163. 10.1016/j.copbio.2016.04.021. 27162093 PMC4975623

[ref105] Moshayedi P.; Nih L. R.; Llorente I. L.; Berg A. R.; Cinkornpumin J.; Lowry W. E.; Segura T.; Carmichael S. T. Systematic Optimization of an Engineered Hydrogel Allows for Selective Control of Human Neural Stem Cell Survival and Differentiation after Transplantation in the Stroke Brain. Biomaterials 2016, 105, 145–155. 10.1016/j.biomaterials.2016.07.028. 27521617 PMC5003628

[ref106] Lam J.; Lowry W. E.; Carmichael S. T.; Segura T. Delivery of IPS-NPCs to the Stroke Cavity within a Hyaluronic Acid Matrix Promotes the Differentiation of Transplanted Cells. Adv. Funct Mater. 2014, 24 (44), 7053–7062. 10.1002/adfm.201401483. 26213530 PMC4512237

[ref107] Ballios B. G.; Cooke M. J.; Donaldson L.; Coles B. L. K.; Morshead C. M.; van der Kooy D.; Shoichet M. S. A Hyaluronan-Based Injectable Hydrogel Improves the Survival and Integration of Stem Cell Progeny Following Transplantation. Stem Cell Reports 2015, 4 (6), 1031–1045. 10.1016/j.stemcr.2015.04.008. 25981414 PMC4471829

[ref108] Payne S. L.; Tuladhar A.; Obermeyer J. M.; Varga B. V.; Teal C. J.; Morshead C. M.; Nagy A.; Shoichet M. S. Initial Cell Maturity Changes Following Transplantation in a Hyaluronan-Based Hydrogel and Impacts Therapeutic Success in the Stroke-Injured Rodent Brain. Biomaterials 2019, 192, 309–322. 10.1016/j.biomaterials.2018.11.020. 30468998

[ref109] Fernández-García L.; Pérez-Rigueiro J.; Martinez-Murillo R.; Panetsos F.; Ramos M.; Guinea G. V.; González-Nieto D. Cortical Reshaping and Functional Recovery Induced by Silk Fibroin Hydrogels-Encapsulated Stem Cells Implanted in Stroke Animals. Front Cell Neurosci 2018, 12, 296 10.3389/fncel.2018.00296. 30237762 PMC6135908

[ref110] Somaa F. A.; Wang T.-Y.; Niclis J. C.; Bruggeman K. F.; Kauhausen J. A.; Guo H.; McDougall S.; Williams R. J.; Nisbet D. R.; Thompson L. H.; Parish C. L. Peptide-Based Scaffolds Support Human Cortical Progenitor Graft Integration to Reduce Atrophy and Promote Functional Repair in a Model of Stroke. Cell Rep 2017, 20 (8), 1964–1977. 10.1016/j.celrep.2017.07.069. 28834757

[ref111] George P. M.; Bliss T. M.; Hua T.; Lee A.; Oh B.; Levinson A.; Mehta S.; Sun G.; Steinberg G. K. Electrical Preconditioning of Stem Cells with a Conductive Polymer Scaffold Enhances Stroke Recovery. Biomaterials 2017, 142, 31–40. 10.1016/j.biomaterials.2017.07.020. 28719819 PMC5575756

[ref112] Balestrino R.; Schapira A. H. V. Parkinson Disease. Eur. J. Neurol 2020, 27 (1), 27–42. 10.1111/ene.14108. 31631455

[ref113] Bloem B. R.; Okun M. S.; Klein C. Parkinson’s Disease. Lancet 2021, 397 (10291), 2284–2303. 10.1016/S0140-6736(21)00218-X. 33848468

[ref114] Kalia L. V.; Lang A. E. Parkinson’s Disease. Lancet 2015, 386 (9996), 896–912. 10.1016/S0140-6736(14)61393-3. 25904081

[ref115] Poewe W.; Seppi K.; Tanner C. M.; Halliday G. M.; Brundin P.; Volkmann J.; Schrag A.-E.; Lang A. E. Parkinson Disease. Nat. Rev. Dis Primers 2017, 3 (1), 17013 10.1038/nrdp.2017.13. 28332488

[ref116] Adil M. M.; Rao A. T.; Ramadoss G. N.; Chernavsky N. E.; Kulkarni R. U.; Miller E. W.; Kumar S.; Schaffer D. V. Dopaminergic Neurons Transplanted Using Cell-Instructive Biomaterials Alleviate Parkinsonism in Rodents. Adv. Funct Mater. 2018, 28 (41), 1804144 10.1002/adfm.201804144.

[ref117] Struzyna L. A.; Browne K. D.; Brodnik Z. D.; Burrell J. C.; Harris J. P.; Chen H. I.; Wolf J. A.; Panzer K. V.; Lim J.; Duda J. E.; España R. A.; Cullen D. K. Tissue Engineered Nigrostriatal Pathway for Treatment of Parkinson’s Disease. J. Tissue Eng. Regen Med. 2018, 12 (7), 1702–1716. 10.1002/term.2698. 29766664 PMC6416379

[ref118] Wang T. Y.; Bruggeman K. F.; Kauhausen J. A.; Rodriguez A. L.; Nisbet D. R.; Parish C. L. Functionalized Composite Scaffolds Improve the Engraftment of Transplanted Dopaminergic Progenitors in a Mouse Model of Parkinson’s Disease. Biomaterials 2016, 74, 89–98. 10.1016/j.biomaterials.2015.09.039. 26454047

[ref119] Rodriguez A. L.; Bruggeman K. F.; Wang Y.; Wang T. Y.; Williams R. J.; Parish C. L.; Nisbet D. R. Using Minimalist Self-Assembling Peptides as Hierarchical Scaffolds to Stabilise Growth Factors and Promote Stem Cell Integration in the Injured Brain. J. Tissue Eng. Regen Med. 2018, 12 (3), e1571-e1579 10.1002/term.2582. 28987031

[ref120] Das S.; Zhou K.; Ghosh D.; Jha N. N.; Singh P. K.; Jacob R. S.; Bernard C. C.; Finkelstein D. I.; Forsythe J. S.; Maji S. K. Implantable Amyloid Hydrogels for Promoting Stem Cell Differentiation to Neurons. NPG Asia Mater. 2016, 8 (9), e304 10.1038/am.2016.116.

[ref121] Nakaji-Hirabayashi T.; Kato K.; Iwata H. In Vivo Study on the Survival of Neural Stem Cells Transplanted into the Rat Brain with a Collagen Hydrogel That Incorporates Laminin-Derived Polypeptides. Bioconjug Chem. 2013, 24 (11), 1798–1804. 10.1021/bc400005m. 23991904

[ref122] Apostolova L. G. Alzheimer Disease. Continuum (Minneapolis, MN) 2016, 22 (2), 419–434. 10.1212/CON.0000000000000307. PMC5390933 27042902

[ref123] Knopman D. S.; Amieva H.; Petersen R. C.; Chételat G.; Holtzman D. M.; Hyman B. T.; Nixon R. A.; Jones D. T. Alzheimer Disease. Nat. Rev. Dis Primers 2021, 7 (1), 33 10.1038/s41572-021-00269-y. 33986301 PMC8574196

[ref124] Ulep M. G.; Saraon S. K.; McLea S. Alzheimer Disease. Journal for Nurse Practitioners 2018, 14 (3), 129–135. 10.1016/j.nurpra.2017.10.014.

[ref125] Lane C. A.; Hardy J.; Schott J. M. Alzheimer’s Disease. Eur. J. Neurol 2018, 25 (1), 59–70. 10.1111/ene.13439. 28872215

[ref126] Cui G.; Shao S.; Yang J.; Liu J.; Guo H. Designer Self-Assemble Peptides Maximize the Therapeutic Benefits of Neural Stem Cell Transplantation for Alzheimer’s Disease via Enhancing Neuron Differentiation and Paracrine Action. Mol. Neurobiol 2016, 53 (2), 1108–1123. 10.1007/s12035-014-9069-y. 25586060 PMC4752586

[ref127] Niemczyk B.; Sajkiewicz P.; Kolbuk D. Injectable Hydrogels as Novel Materials for Central Nervous System Regeneration. J. Neural Eng. 2018, 15 (5), 051002 10.1088/1741-2552/aacbab. 29889043

[ref128] Samadian H.; Maleki H.; Fathollahi A.; Salehi M.; Gholizadeh S.; Derakhshankhah H.; Allahyari Z.; Jaymand M. Naturally Occurring Biological Macromolecules-Based Hydrogels: Potential Biomaterials for Peripheral Nerve Regeneration. Int. J. Biol. Macromol. 2020, 154, 795–817. 10.1016/j.ijbiomac.2020.03.155. 32198035

[ref129] Brull R.; Hadzic A.; Reina M. A.; Barrington M. J. Pathophysiology and Etiology of Nerve Injury Following Peripheral Nerve Blockade. Reg Anesth Pain Med. 2015, 40 (5), 479–490. 10.1097/AAP.0000000000000125. 25974275

[ref130] Nisbet D. R.; Crompton K. E.; Horne M. K.; Finkelstein D. I.; Forsythe J. S. Neural Tissue Engineering of the CNS Using Hydrogels. J. Biomed Mater. Res. B Appl. Biomater 2008, 87B (1), 251–263. 10.1002/jbm.b.31000. 18161806

[ref131] Aijie C.; Xuan L.; Huimin L.; Yanli Z.; Yiyuan K.; Yuqing L.; Longquan S. Nanoscaffolds in Promoting Regeneration of the Peripheral Nervous System. Nanomedicine 2018, 13 (9), 1067–1085. 10.2217/nnm-2017-0389. 29790811

[ref132] Carriel V.; Garrido-Gómez J.; Hernández-Cortés P.; Garzón I.; García-García S.; Sáez-Moreno J. A.; del Carmen Sánchez-Quevedo M.; Campos A.; Alaminos M. Combination of Fibrin-Agarose Hydrogels and Adipose-Derived Mesenchymal Stem Cells for Peripheral Nerve Regeneration. J. Neural Eng. 2013, 10 (2), 026022 10.1088/1741-2560/10/2/026022. 23528562

[ref133] Chato-Astrain J.; Campos F.; Roda O.; Miralles E.; Durand-Herrera D.; Sáez-Moreno J. A.; García-García S.; Alaminos M.; Campos A.; Carriel V. In Vivo Evaluation of Nanostructured Fibrin-Agarose Hydrogels With Mesenchymal Stem Cells for Peripheral Nerve Repair. Front Cell Neurosci 2018, 12, 501 10.3389/fncel.2018.00501. 30627086 PMC6309160

[ref134] Meyer C.; Wrobel S.; Raimondo S.; Rochkind S.; Heimann C.; Shahar A.; Ziv-Polat O.; Geuna S.; Grothe C.; Haastert-Talini K. Peripheral Nerve Regeneration through Hydrogel-Enriched Chitosan Conduits Containing Engineered Schwann Cells for Drug Delivery. Cell Transplant 2016, 25 (1), 159–182. 10.3727/096368915X688010. 25876520

[ref135] Gonzalez-Perez F.; Hernández J.; Heimann C.; Phillips J. B.; Udina E.; Navarro X. Schwann Cells and Mesenchymal Stem Cells in Laminin- or Fibronectin-Aligned Matrices and Regeneration across a Critical Size Defect of 15 Mm in the Rat Sciatic Nerve. J. Neurosurg Spine 2018, 28 (1), 109–118. 10.3171/2017.5.SPINE161100. 29125428

[ref136] Salehi M.; Bagher Z.; Kamrava S. K.; Ehterami A.; Alizadeh R.; Farhadi M.; Falah M.; Komeili A. Alginate/Chitosan Hydrogel Containing Olfactory Ectomesenchymal Stem Cells for Sciatic Nerve Tissue Engineering. J. Cell Physiol 2019, 234 (9), 15357–15368. 10.1002/jcp.28183. 30701533

[ref137] Demyanenko S. V.; Pitinova M. A.; Kalyuzhnaya Y. N.; Khaitin A. M.; Batalshchikova S. A.; Dobaeva N. M.; Shevtsova Y. A.; Goryunov K. V.; Plotnikov E. Y.; Pashkevich S. G.; Sukhikh G. T.; Silachev D. N. Human Multipotent Mesenchymal Stromal Cell-Derived Extracellular Vesicles Enhance Neuroregeneration in a Rat Model of Sciatic Nerve Crush Injury. Int. J. Mol. Sci. 2022, 23 (15), 8583 10.3390/ijms23158583. 35955732 PMC9369448

[ref138] Sun Y.; Li W.; Wu X.; Zhang N.; Zhang Y.; Ouyang S.; Song X.; Fang X.; Seeram R.; Xue W.; He L.; Wu W. Functional Self-Assembling Peptide Nanofiber Hydrogels Designed for Nerve Degeneration. ACS Appl. Mater. Interfaces 2016, 8 (3), 2348–2359. 10.1021/acsami.5b11473. 26720334

[ref139] Mobarakeh Z. T.; Hasanzadeh E.; Farzin A.; Goodarzi A.; Farahani M. S.; Shirian S.; Mahmoodi N.; Zamani N.; Karimi A.; Ai J. Enhanced Sciatic Nerve Regeneration with Fibrin Scaffold Containing Human Endometrial Stem Cells and Insulin Encapsulated Chitosan Particles: An in Vivo Study. Injury 2023, 54 (6), 1462–1472. 10.1016/j.injury.2023.01.041. 36894467

